# Evolutionary and Ecological Drivers Shape the Emergence and Extinction of Foot-and-Mouth Disease Virus Lineages

**DOI:** 10.1093/molbev/msab172

**Published:** 2021-06-11

**Authors:** Antonello Di Nardo, Luca Ferretti, Jemma Wadsworth, Valerie Mioulet, Boris Gelman, Sharon Karniely, Alexey Scherbakov, Ghulam Ziay, Fuat Özyörük, Ünal Parlak, Pelin Tuncer‐Göktuna, Reza Hassanzadeh, Mehdi Khalaj, Seyed Mohsen Dastoor, Darab Abdollahi, Ehtisham-ul-Haq Khan, Muhammad Afzal, Manzoor Hussain, Nick J Knowles, Donald P King

**Affiliations:** 1 The Pirbright Institute, Ash Road, Pirbright, Woking, Surrey, United Kingdom; 2 Big Data Institute, Li Ka Shing Centre for Health Information and Discovery, Nuffield Department of Medicine, University of Oxford, Oxford, United Kingdom; 3 Division of Virology, Kimron Veterinary Institute, Beit Dagan, Israel; 4 Federal Governmental Budgetary Institution “Federal Centre for Animal Health” (FGBI “ARRIAH”), Yur’evets, Vladimir, Russia; 5 Central Veterinary Diagnostic and Research Laboratory, Kabul, Afghanistan; 6 Faculty of Veterinary Medicine, Harran University, Sanliurfa, Turkey; 7 Foot and Mouth Disease (ŞAP) Institute, Ankara, Turkey; 8 Iran Veterinary Organization, Ministry of Jihad-e-Agriculture, Tehran, Iran; 9 Livestock and Dairy Development Department, Government of Punjab, Rawalpindi, Pakistan; 10 Food and Agriculture Organization of the United Nations, Pakistan Office, Islamabad Pakistan

**Keywords:** foot-and-mouth disease, phylogeography, phylodynamics, Western, Central, and Southern Asia, molecular epidemiology

## Abstract

Livestock farming across the world is constantly threatened by the evolutionary turnover of foot-and-mouth disease virus (FMDV) strains in endemic systems, the underlying dynamics of which remain to be elucidated. Here, we map the eco-evolutionary landscape of cocirculating FMDV lineages within an important endemic virus pool encompassing Western, Central, and parts of Southern Asia, reconstructing the evolutionary history and spatial dynamics over the last 20 years that shape the current epidemiological situation. We demonstrate that new FMDV variants periodically emerge from Southern Asia, precipitating waves of virus incursions that systematically travel in a westerly direction. We evidence how metapopulation dynamics drive the emergence and extinction of spatially structured virus populations, and how transmission in different host species regulates the evolutionary space of virus serotypes. Our work provides the first integrative framework that defines coevolutionary signatures of FMDV in regional contexts to help understand the complex interplay between virus phenotypes, host characteristics, and key epidemiological determinants of transmission that drive FMDV evolution in endemic settings.

## Introduction

The livestock sector has expanded rapidly over the past decades, particularly in low- and middle-income countries (LMICs), where the increasing demand for livestock products has been driven by economic and population growth, rising per capita income, and urbanization. Traditional livestock management systems have evolved to optimize the use of resources, and have been transformed by the implementation of more intensive farming units overlaid on top of more traditional small-scale systems (Thornton 2010). Although enhanced livestock markets offer access to better nutrition and many economic opportunities, a less sustainable-oriented intensification of farming systems can also lead to a greater potential for pathogen emergence and spread. As a consequence, the modernization of farming systems brings new challenges in terms of efficient management of animal health risks. Thus, a better understanding of the epidemiological consequences in ecosystems modified by human-driven changes is required in order to ultimately improve the management of infectious diseases and reduce (and ultimately eliminate) their impacts on societies, economies, and political systems (Cleaveland et al. 2017). 

From an economic perspective, the most damaging epidemics are caused by pathogens of animal origin, and increasingly those of zoonotic concern as our environment changes (Karesh et al. 2012; Jones et al. 2013; Hassell et al. 2017), or where multiple host species are affected with the potential of being readily transmitted at wide geographical scales (Cleaveland et al. 2001). Globally, foot-and-mouth disease (FMD) is an important disease of livestock due to its direct impact on livestock productivity and enormous economic consequences on the trade of animals and animal products. Such impacts are particularly severe in LMICs where livestock is a fundamental component of the agricultural-based economy and social structure of people, with production losses due to endemic FMD estimated at between US$1.2 and 2.3 billion with over 50 million of animals affected every year (Knight-Jones and Rushton 2013). In traditional livestock-dependent communities, FMD threatens livestock productivity, food security, and rural income generation, as well as decreasing local market opportunities. At the international level, FMD is a major barrier to trade, preventing the export of livestock and their products to more profitable markets found in developed countries. Although there is a clear need to address these challenges through well-designed control policies, controlling FMD is a very expensive exercise and is often not considered as a priority in LMICs (Fukase 2012).

In Western and Southern Asia, significant challenges are represented by persistent outbreaks due to multiple serotypes and strains of the FMD virus (FMDV) that result in high levels of disease endemicity. Across this largely interconnected ecosystem and epidemiological reservoir, which defines the extent of the FMDV endemic Pool 3 (Paton et al. 2009), FMD epidemiology is characterized by waves of infection sustained by newly emerging FMDV variants supplanting older lineages (Arrowsmith 1975; Ansell et al. 1994; Knowles et al. 2005, 2009; Valarcher et al. 2009). These epizooties (such as those historically caused by: serotype O ME-SA/PanAsia and PanAsia-2 lineages; serotype A ASIA/A_22_, Iran-96, Iran-99, and Iran-05 lineages; and serotype Asia1) are representative of FMD viruses that have been adapted and evolved within and beyond their ecosystems of origin, leading to rapid international spread. Clear examples of how enzootic FMDV strains, emerging from Southern Asia and initially characterized by a very limited geographical distribution, can rapidly spread and readily progress toward a panzootic status have been previously documented with the incursion of the type O PanAsia-2 lineage into Bulgaria during 2010–2011 (Valdazo-Gonzalez et al. 2012), and the global spread of the serotype O ME-SA/Ind-2001 lineage (Bachanek-Bankowska et al. 2018). The coexistence of livestock systems of both intensive and small-scale nature, the presence of informal routes for transboundary trade in livestock, a lack of proper health certifications for live animal trade, and the absence of properly implemented disease prevention programs are some of the likely key drivers at the core of the current epidemiological challenge.

The evolution of pathogens is determined by the close interplay between ecological and epidemiological factors, with RNA viruses like FMDV evolving rapidly, leaving footprints of their genetic variation observable in epidemiological time. Phylogenetic frameworks that integrate trait, as well as covariates of epidemiological and evolutionary processes, with sequence evolution into pathogen phylodynamics have been formalized (Baele et al. 2017) and used to study and characterize transmission dynamics of viral epidemics and emerging diseases (Faria et al. 2014; Dudas et al. 2017; Grubaugh et al. 2017). Molecular epidemiological studies performed using FMDV sequence data have helped to understand the spatiotemporal dynamics of single virus lineages (Hall et al. 2013; Di Nardo et al. 2014; Bachanek-Bankowska et al. 2018), epidemic incursions in previously unaffected areas (Valdazo-Gonzalez et al. 2012), dynamics of serotypes circulation in Africa (Lycett et al. 2019; Gizaw et al. 2020), and patterns of between- and within-host recombination (Bachanek-Bankowska et al. 2018; Ferretti et al. 2018; Lasecka-Dykes et al. 2018). FMD circulation in endemic systems is characterized by complex dynamics of coexisting viral lineages evolving within and between distinct ecological systems, with transmission likely to be influenced by host factors, such as viral fitness in different hosts, pre-existing immunity, and livestock mobility. However, the processes that drive evolutionary fitness of FMDV populations from emergence to expansion in endemic systems, their geographical structure, as well as the evolutionary dynamics on host populations that contribute to virus adaptation and persistence are not well understood.

This study synthesizes information collected over 20 years and provides the first integrative framework that investigates coevolutionary signatures of FMDV in a regional context by describing the activity of the most diverse and persistent FMDV strains circulating within the FMDV Endemic Pool 3 (Paton et al. 2009), namely the O/ME-SA/PanAsia-2, A/ASIA/Iran-05, and Asia1/ASIA/Sindh-08 FMDV lineages. By analyzing a total of 2,495 FMDV sequences encoding the VP1/1D protein generated from clinical samples collected between 2001 and 2018, we have mapped the history and dispersal of these FMDV lineages across Western and Southern Asia, defining the geographical structure of virus metapopulations and their source-sink dynamics on an evolutionary scale. Building on the understanding of the spatial dynamics of coexisting FMDV lineages, we also investigated virus–host interactions and host-specific contributions to the evolutionary mode of FMDV transmission, describing how serotype-specific fitness on domesticated livestock is mediated by host characteristics and their distributions. We further determined geographic, population, economic, and trade factors that likely influence patterns of FMDV transmission and contribute to the spread of the virus at a regional level and beyond. Being the largest study of its kind undertaken for FMD to date, we anticipate that the novel insights into the evolutionary dynamics of FMDV presented here for one of the largest and most complex FMD endemic systems will help to tailor prevention and intervention strategies that will better guide the global control of FMD.

## Results

### Evolutionary Clock and Selection of FMDV

**Fig. 1.**
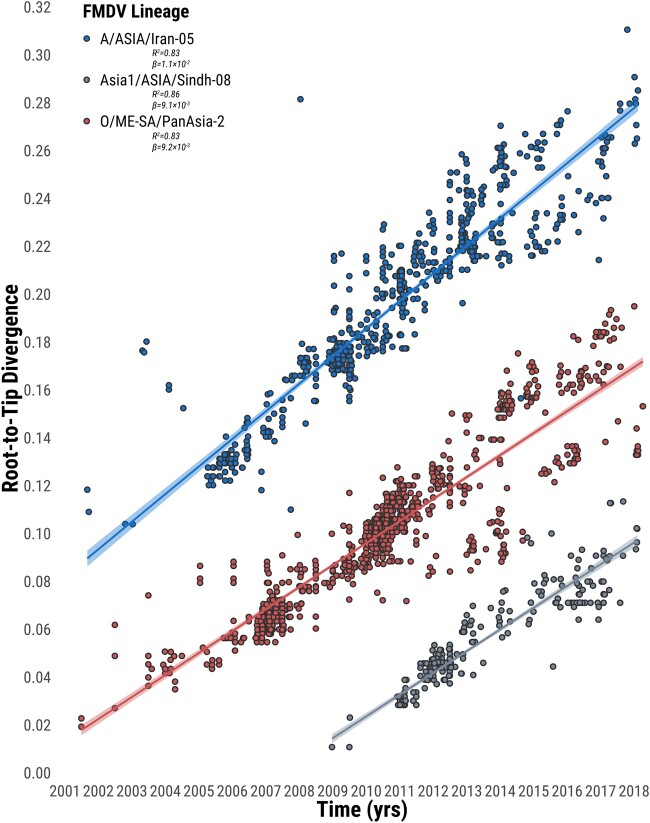
Linear regression of sample collection dates against divergence from the root of reconstructed maximum-likelihood trees from FMDV VP1 sequences. Circles representing tip nodes are colored according to the corresponding FMDV lineage. Fitted lines are shown with their estimated 95% confidence interval regions. *R*-squared (*R*^2^) and slope (*β*) parameters estimated for each fitted regression line are reported.

We compiled an extensive data set of FMDV VP1/1D coding sequences (*n* = 2,495) comprising of O/ME-SA/PanAsia-2 (*n* = 1,321), A/ASIA/Iran-05 (*n* = 843), and Asia1/ASIA/Sindh-08 (*n* = 331) FMDV lineages that were contemporarily circulating in Western (*n* = 648), Southern Asia (*n* = 1,720), and bordering regions (*n* = 127) during the period between 2001 and 2018. Analyses of the root-to-tip distances estimated from the maximum-likelihood trees as a function of the sampling time revealed a strong temporal signal of FMDV evolution (average *R*^2^ of 0.84) (fig. 1). FMDV lineages were found to evolve at a very similar rate, estimated at an average of 1.3 × 10^−2^ nucleotide substitutions/site/year (min–max range of 1.1 × 10^−2^–1.4 × 10^−2^), with some variation reported across branches (min–max range for standard deviation of rates 1.3 × 10^−2^–2.3 × 10^−2^) (table 1). These results indicate that FMDV evolves under a strict molecular clock that is largely constant among the different serotypes, irrespective of the diverse path of evolutionary histories characterizing genome variants.

**Table 1.** Evolutionary and Population Genetics Parameters Estimated for Each of the FMDV Lineages.

	A/ASIA/Iran-05	O/ME-SA/PanAsia-2	Asia1/ASIA/Sindh-08
tMRCA	1997.141 [1,994.068–1.999.687]	1.999.669 [1,997.869–2,001.094]	2,008.757 [2,008.164–2,008.978]
Evolution rate	1.41 × 10^−2^ [1.23 × 10^−2^–1.58 × 10^−2^]	1.15 × 10^−2^ [9.95 × 10^−3^–1.33 × 10^−2^]	1.22 × 10^−2^ [9.23 × 10^−3^–1.53 × 10^−2^]
Evolution rate SD	1.30 × 10^−2^ [1.02 × 10^−2^–1.61 × 10^−2^]	1.13 × 10^−2^ [8.67 × 10^−3^–1.43 × 10^−2^]	2.29 × 10^−2^ [1.25 × 10^−2^–3.62 × 10^−2^]
Coef. of variation	0.90 [0.75–1.06]	0.96 [0.81–1.10]	1.55 [1.11–2.10]
Covariance	−9.48 × 10^−3^ [−5.50 × 10^−2^–3.54 × 10^−2^]	−3.69 × 10^−3^ [−3.91 × 10^−2^–3.51 × 10^−2^]	−5.70 × 10^−3^ [−6.72 × 10^−2^–6.72 × 10^−2^]
Migration rate	8.63 × 10^−2^ [6.66 × 10^−2^–1.09 × 10^−1^]	8.84 × 10^−2^ [6.80 × 10^−2^–1.10 × 10^−1^]	6.81 × 10^−2^ [3.48 × 10^−2^–1.04 × 10^−1^]
Dispersal velocity	738.7 [701.1–770.4]	734.3 [716.8–761.4]	647.5 [601.3–689.1]
Diffusion rate	159,965.2 [149,277.5–168,128.2]	178,453.3 [173,145.5–185,131.3]	158,029.3 [144,489.2–186,222.9]
α-diversity	1.906	2.623	1.789
β-diversity	2.415	1.130	–
*F*_ST_	0.559	0.301	–

Note.—Parameter values are reported as posterior mean with their associated 95% BCI. Evolutionary rate is defined as “nucleotide substitutions/site/year,” migration rate as “migration events/year,” dispersal velocity in “km/year,” and diffusion rate in “km^2^/year.”

Analyses of selective pressure on sequences encoding the VP1/1D protein revealed a higher estimated global *d_N_*/*d_S_* ratio for A/Iran-05, compared with the other two viral lineages (supplementary table S1 and fig. S1*a*, Supplementary Material online). A total of nine, three, and ten amino acid (AA) sites of the VP1/1D protein were identified to be under significant positive selection (PP ≥ 0.9) for A/Iran-05, O/PanAsia-2, and Asia1/Sindh-08, respectively (supplementary table S1, Supplementary Material online). The locations of some of these AA sites on the external structure of the FMDV were found to be shared by all FMDV lineages (residues 140–142, flanking the conserved RGD motif and included within βG-βH loop), whilst other residues were shared only between A/Iran-05 and Asia1/Sindh-08 (residues 43–45, part of the βB-βC loop) or between A/Iran-05 and O/PanAsia-2 (residues 196–197, within the VP1 c-terminal region following beta-sheet I) (supplementary fig. S1*b* and *c*, Supplementary Material online). These three regions of VP1/1D, representing antigenic sites 1 and 3, have previously been identified as containing antigenically important AA residues critical for virus neutralization and to initiate infection (Thomas et al. 1988; Bolwell et al. 1989; Kitson et al. 1990; Saiz et al. 1991; Grazioli et al. 2013).

### FMDV Transmission and Dispersal at Regional Level

**Fig. 2.**
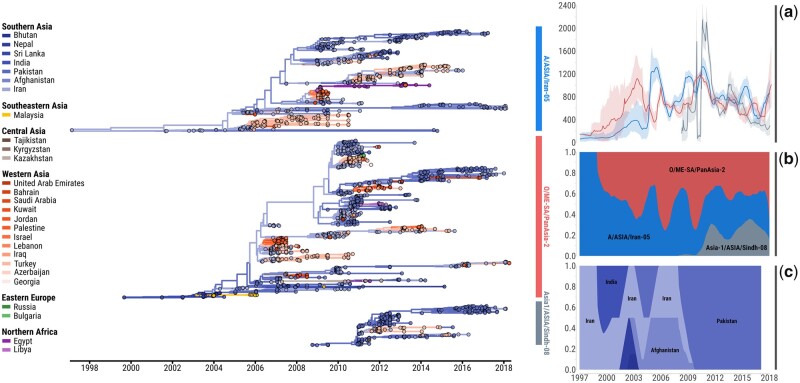
Space-time phylodynamics of FMDV lineages circulating in Western and Southern Asia between 1997 and 2018. In the left panel, combined maximum clade credibility (MCC) tree reconstructed for each of the FMDV lineages investigated. Branches and nodes are colored according to the most probable ancestral location inferred from the discrete phylogeography analysis. (*a*) Evolution of the weighted dispersal velocity (*y*-axis, expressed in km/year) through time (*x*-axis) estimated using continuous phylogeography methods and reconstructed for each of the FMDV lineages. (*b*) Inferred FMDV lineage ancestry makeup (*y*-axis) through time (*x*-axis) estimated from the combined MCC tree topologies. (*c*) Proportion of ancestral locations of FMDV lineages (*y*-axis) through time (*x*-axis) extracted from the combined phylogenetic tree trunks as inferred from the discrete phylogeographic analysis. The plots were reconstructed using the data used from this study, and they do not reflect FMDV lineages that were circulating and sampled prior to 2001.

**Fig. 3.**
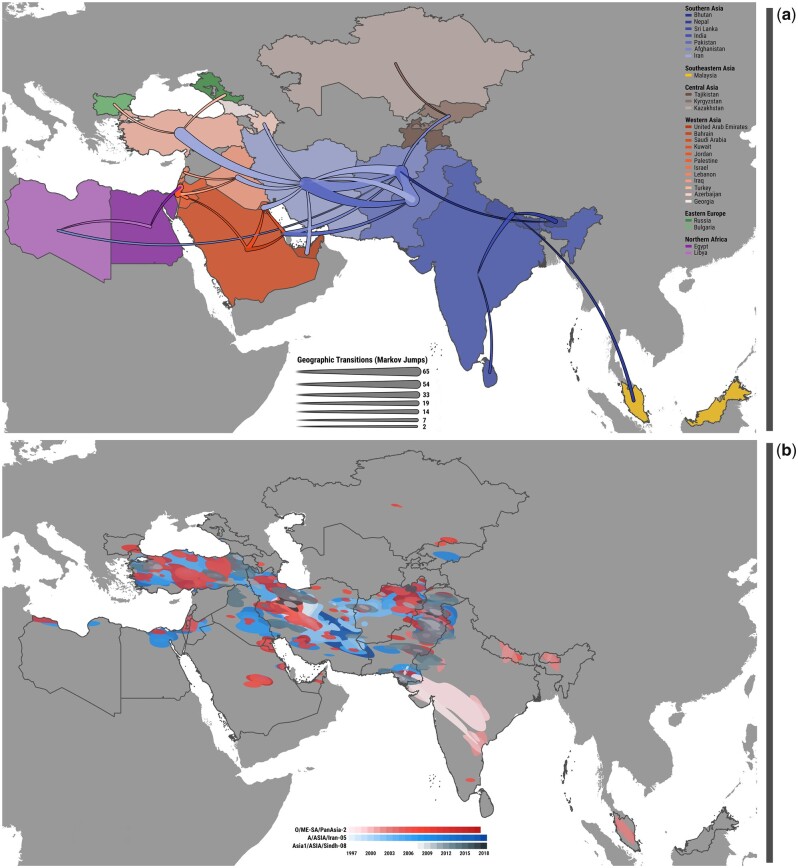
Spatial dynamics of FMDV dispersal within Western and Southern Asia. (*a*) Size and directionality (expressed from the thin to the thick end) of discrete phylogeographic reconstructed migrations of FMDV lineages between countries (expressed by their centroids) are represented with Bezier curves, with their size proportional to the median number of Markov jumps inferred from the discrete phylogeographic analysis and summed across all three FMDV lineages. (*b*) Continuous phylogeographic diffusion in space and time showing the 95% highest posterior density (HPD) contours (kernel density estimates) computed for each FMDV lineages and sequentially colored by year with gradients ranging from light (time of the most recent common ancestor) to dark (time of the most recent sample). The HPD regions were derived from 100 trees uniformly sampled from the posterior space of the continuous phylogeographic model results.

By reconstructing ancestral diffusion of FMDV at the regional scale using discrete phylogeography, we identified tree-trunk viruses (which represent those ancestral lineages that persist in time across generations) as solely originating from Southern Asian countries (fig. 2*a* and *c*), demonstrating the importance of this region as a constant source of evolutionary novelty for emerging FMDV variants. Specifically, these analyses highlight the pivotal role played by virus circulation in Pakistan, Iran, and Afghanistan (proportion of time in the trunk of 0.44, 0.30, and 0.11, respectively), supporting the idea that these countries represent primary conveyors of FMDV infection across the region and are important sites for generating genomic diversity. Discrete phylogeographic analysis was used to map the structure and connectivity of the FMDV migration network (figs. 2*a* and 3*a*, supplementary movies S1–S3, Supplementary Material online), showing how viruses diffuse across the region along well-defined transmission routes, with movements of viruses in a westerly direction accounting for 60% of the full geographic transitions reconstructed from the evolutionary history of all FMDV lineages. Phylogeographic reconstructions further revealed three main patterns of transboundary movements of viruses (fig. 3*a*, supplementary movies S1–S3, Supplementary Material online): 1) the continuous virus interchange between Pakistan, Afghanistan, and Iran (sum of median Markov jumps: 170), likely constituting interconnected large metapopulations that favor virus exchange and persistence; 2) the key role of Iran as hub of virus diffusion to the west (sum of median Markov jumps: 52), possibly acting as a habitat corridor for virus dispersal between the Southern Asia region and countries further to the west; and 3) the unidirectional migration of viruses from Iran toward Turkey (sum of median Markov jumps: 36). These transmission pathways represent, respectively, 61%, 19%, and 13% of all geographic transitions recorded within the region. An important observation was that this spatial network of virus transmission was found to be shared by all FMDV serotypes, which were thus reported to circulate at the very same geographical scale. More detailed timelines of geographic transitions reconstructed by the discrete phylogeographic analyses and describing the FMDV virus diffusion across the Endemic Pool 3 for each of the lineages examined are provided as supplementary text S1, Supplementary Material online.

Viruses were estimated to flow across the entire region at an average rate of 0.08 (95% Bayesian credible Intervals [BCI] 0.05–0.11) transboundary migration events per lineage per year, with similar rates reported for the A/Iran-05 and O/PanAsia-2 lineages, but lower for the Asia1/Sindh-08 lineage (table 1). Average rates of more than two transboundary migration events per lineage per year were recorded within the evolutionary ecosystem of the Southern Asia region (again comprised by Afghanistan, Pakistan, and Iran), and also from the latter to Turkey. By analyzing the results of the continuous space phylogeography model, we further observed rapid movement of viruses to the west with the epizootic wavefront of FMDV moving on average at a speed of ∼730 km/year for the A/Iran-05 and O/PanAsia-2 lineages (table 1), whereas the Asia1/Sindh-08 lineage was not so widely dispersed in the region.

### Evolutionary Dynamics of Cocirculating FMDV Lineages

Evolutionary trajectories of FMDV populations revealed cyclical dynamics of alternating serotypes, with infections sustained by single lineages predominating at time intervals (fig. 2*b*), a trend that was further evidenced by plotting the historical changes in viral diversity through time (supplementary fig. S2, Supplementary Material online). From 2002 onwards, we estimated a periodicity in the predominance of individual viral lineages at about 3 years. As evidenced by discrete phylogeographic reconstruction, the seeding frequency of trunk virus populations was found to be geographically structured and followed an oscillatory pattern of infections (fig. 2*c*), indicating the likely evolutionary sources for each of the FMDV lineages. For A/Iran-05 and O/PanAsia-2, these ancestral viruses were geographically located across the Southern Asia region (with the earliest ancestry of O/PanAsia-2 originating within the Indian subcontinent, and from Iran for A/Iran-05), whilst the Asia1/Sindh-08 lineage originated from Pakistan. From 2010 onwards, Pakistan was identified as the dominant trunk location of ancestral virus populations for all lineages.

Analyzing the migratory patterns of FMDV in space and time using the continuous diffusion model (fig. 3*b*), we found that viral lineages were characterized by high spatial diffusivity ($D_{w}\;$= ∼180,000 km^2^/year, which corresponded to a wavefront moving at ∼400 km in a year or about 1 km per day) with slightly higher estimates obtained for the O/PanAsia-2 lineage (table 1), although this difference was not found to be statistically significant. It was also evident that the diffusivity of FMDV lineages was highly variable (supported by the relatively large coefficient of variation of $D_{w}$ among branches), due to the presence of tree branches representing long-distance displacements of viruses. In addition, statistics of dispersal velocity estimated from the continuous phylogeographic data for each of the FMDV lineages revealed a remarkable variability in the spatial dynamics of FMDV lineage expansion across the region (fig. 2*a*). Increases in the velocity of viral dispersal for a given FMDV serotype were observed to parallel, in relative time, with reduction in the dispersal velocity of the other serotypes in a dynamical alternating fashion. We hypothesize that stochastic fluctuations in timing and relative prevalence of different FMDV lineages are responsible for initiating the observed waves of alternating serotypes that, once established, are maintained by spatial competition, pre-existing immunity patterns generated by previous waves, and sequential replacement of specific viruses occupying favorable ecological niches. It is worth noting that this spatial tendency of serotypes dispersal matched the cyclical dynamics of infection by alternating serotypes (as previously described), supporting a positive correlation between the speed at which FMDV infections spread across the region and the frequency of FMDV lineages observed at the phylogenetic level, as expected from traveling waves above a background of low endemic FMD prevalence (fig. 2*a* and *b*, supplementary fig. S2, Supplementary Material online).

### Emergence and Spatial Structure of FMDV Sublineages

**Fig. 4.**
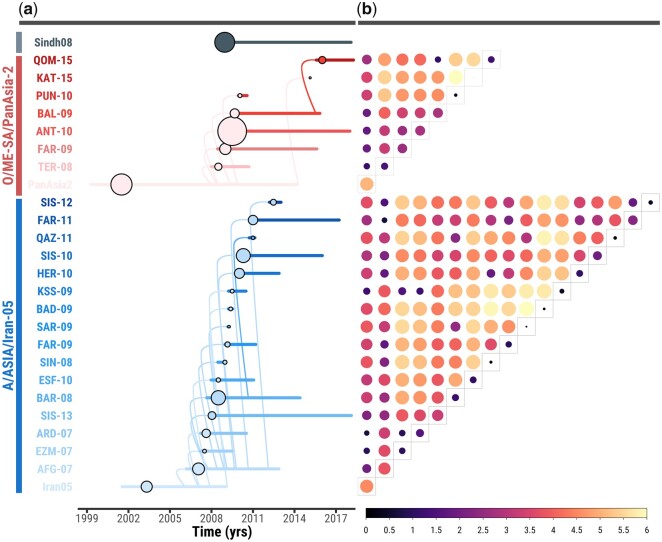
Ancestral evolution and genetic diversity of FMDV sublineages circulating in Western and Southern Asia. (*a*) Horizontal lines represent the evolutionary duration of each FMDV sublineage from the time of the first to the time of the last sampled isolate (the individual dot for PanAsia-2^KAT-15^ denotes a single report). Ancestral links between FMDV sublineages are represented by arches, with their starting point denoting the time of the most recent common ancestor (tMRCA). The area of circles is indicative of the number of sequences present for each FMDV sublineage. (*b*) Between- (β) and within- (α) sublineage diversity is expressed using the Rao’s quadratic entropy, with the size of the circle proportional to the estimated entropy value. Estimates of α-diversity are represented by circles present in the diagonal of the correlogram.

FMDV lineages detected in the region continuously evolved during the timeline of the study to generate new FMDV sublineages, where a >5% genetic divergence cutoff in similarity matrices of most closely related FMDV prototypes was used to define the presence of a new viral sublineage (Knowles and Samuel 2003). The emergence of distinct virus sublineages was characterized within both the A/Iran-05 and O/PanAsia-2 FMDV strains recording, respectively, 17 and 8 different sublineages having circulated from 2006 onwards (fig. 4*a*). Phylogenetic tree topologies representing the relationship between these FMDV sublineages were variable, some presented as a ladder-like topology indicative of a continuous evolutionary turnover, whilst others comprised separately grouped viruses suggestive of spatial mixing.

For A/Iran-05, the evolutionary origin of new sublineages was dominated by two FMDV sublineages: the initial Iran-05, which persisted on the trunk until late-2005 and became the progenitor of 6 of the other 16 sublineages, subsequently replaced by the Iran-05^AFG-07^ from early 2006 until mid-2009, which in turn seeded 9 sublineages (fig. 4*a*, supplementary text S1, Supplementary Material online). Regarding the evolution of O/PanAsia-2, we found that six out of the other seven sublineages originated from the initial PanAsia-2, which was found to be the dominant sublineage observed at the tree-trunk level until mid-2009. This was subsequently replaced by the monophyletic PanAsia-2^ANT-10^, which did not generate any further new sublineages (fig. 4*a*, supplementary text S1, Supplementary Material online). The degree of differentiation among FMDV lineages was estimated to be higher for the A/Iran-05 (*F_ST_* = 0.56; *γ*-diversity = 4.32) that showed higher diversity between sublineages evolving from the ancestral Iran-05 (*β*-diversity = 2.41), which likely indicates continuous adaptation with high turnover of FMDV sublineages (fig. 4*b*, table 1). In contrast, FMDV sublineages evolving within the O/PanAsia-2 lineage clade were characterized by less differentiation from the ancestral PanAsia-2 (*F_ST_* = 0.30; *β*-diversity = 1.13), although a higher diversity was observed at the within-sublineage level (*α*-diversity = 2.62), suggestive of selective sweep processes, which was further supported by the close distance in time of the tMRCAs estimated from the PanAsia-2 tree trunk for each sublineage (fig. 4*b*, table 1).

By analyzing the distribution of FMDV sublineages reconstructed in continuous space, we detected multiple sublineages cocirculating as geographically structured populations (supplementary figs. S4 and S5, Supplementary Material online), with patterns of virus diffusion found to be consistent across different serotypes. Some sublineages were characterized by a highly diffusive nature, emerging locally and rapidly expanding across the entire region at an epizootic level, thus likely generating sustained infections among a large host population size (as observed for PanAsia-2^ANT-10^ and Iran-05^BAR-08^) (supplementary figs. S4 and S5, Supplementary Material online). In contrast, other sublineages were more geographically restricted to specific areas, with some located solely within Western Asia (such as PanAsia-2^QOM-15^ and Iran-05^QAZ-11^) (supplementary figs. S4 and S5, Supplementary Material online) or Southern Asia (reported for PanAsia-2^BAL-09^ and Iran-05^FAR-11^) (supplementary figs. S4 and S5, Supplementary Material online). Certain sublineages, originating in north-west Iran and then diffusing into Turkey, were characterized by spatial dead-end transmissions while continuing to genetically evolve in Turkey (e.g. PanAsia-2^TER-08^ and Iran-05^ARD-07^) (supplementary figs. S4 and S5, Supplementary Material online). Despite observing a general correlation between the duration and distance of spatial dispersal, some sublineages were characterized by low diffusivity with limited geographical expansion (such as PanAsia-2^PUN-10^ circulating only in areas of Pakistan and Iran-05^SAR-09^ in some of Afghanistan) (supplementary figs. S4 and S5, Supplementary Material online). These findings indicate that the epidemiology of certain FMDV sublineages was geographically restricted, likely involving only local transmission in host populations as they move within their typical home ranges. Statistics of virus diffusivity further confirmed heterogeneity in the dispersal velocity of both A/Iran-05 and O/PanAsia-2 sublineages that spatially characterized their migration across the region (supplementary table S2 and fig. S3*a* and *b*, Supplementary Material online). These patterns of virus diffusion are highly indicative of a metapopulation model of the directional flow of spatially heterogeneous and temporally varying FMDV populations.

### Virus-Host Fitness and Impact on Virus Transmission

**Fig. 5.**
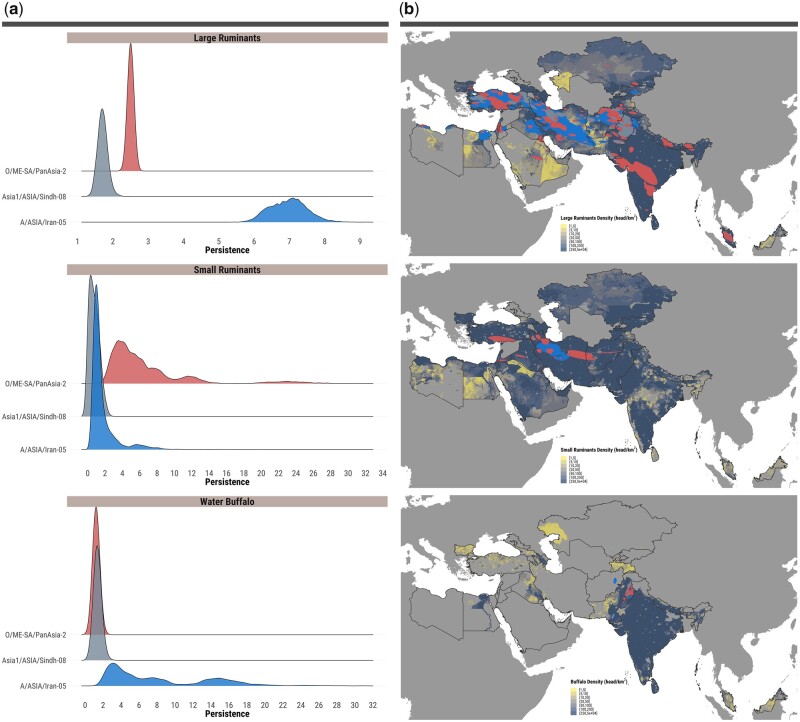
Dynamics of FMDV lineages in host species within Western and Southern Asia. (*a*) Posterior distribution of evolutionary persistence of FMDV lineages in different host species obtained from the structured coalescent analysis. Evolutionary persistence is expressed as a measure of the waiting time (in years) for a datum FMDV lineage to be transmitted between different host species. (*b*) Kernel density estimates of FMDV lineages dispersal within single host species superimposed to the population density of host species distribution (expressed in heads/km^2^) defined within the boundaries of Western, Central, and Southern Asia. The 95% highest posterior density (HPD) regions were derived from 100 trees uniformly sampled from the posterior space of the structured coalescent analysis and aggregated to the continuous phylogeographic model results. The spatial raster of population density for each host species was sourced from the FAO Gridded Livestock of the World (Gilbert et al. 2018).

We estimated that FMDV infections due to interhost species transmissions occurred at an average rate of 0.13 events per year (95% BCI 0.09–0.17). We further observed statistical differences between FMDV lineages in the rate of cross-species transmission (*F*_2,15,781_ = 162,300, *P *<* *0.001), with higher rates reported for the Asia1/Sindh-08 lineage and lower for the A/Iran-05 lineage. By jointly analyzing the continuous estimates of FMDV spatial diffusion with the results of the structured coalescent analysis of host populations, the important contribution of cattle to the epizootic expansion was revealed at both the within- and between-species levels (total number of median host transitions of 4,198 and 270, respectively) (fig. 5*b*). In contrast, water buffalo (*Bubalus bubalis*) only contributed to these epidemiological dynamics at a fine level, as revealed by the more limited geographical clustering of within-buffalo transmissions (total number of median host transitions of 361) observed in two regions of Pakistan, the Punjab and Sindh (fig. 5*b*). FMDV transmission within small ruminant populations also appeared to be geographically clustered, restricted to discrete areas in Southern and Western Asia: the central and western regions of Iran; the border regions between Iran and Afghanistan (i.e. Khorasan and Farah/Herat); Israel and Palestine; and southern-central Anatolian Turkey (fig. 5*b*). It is further interesting to note that the evolutionary persistence of FMDV in different hosts varied according to the transmitting serotype (fig. 5*a*, supplementary table S3, Supplementary Material online). Here we define persistence as a measure of cross-species transmission (i.e. the waiting time, in years, for a virus to jump to a new host population), as a proxy estimate of the diversity FMDV is accumulating by evolving within a specific host species. Waiting time for virus transfer between hosts was observed to be longer in large ruminants infected by A/Iran-05 viruses, different from the O/PanAsia-2 lineage which showed a markedly shorter duration in cattle (median difference in evolutionary persistence between serotype A and O lineages of 4.5 years) (fig. 5*a*, supplementary table S3, Supplementary Material online). A contrasting pattern was reported for small ruminants, where the waiting time for interspecies transmission was longer in O/PanAsia-2 infected populations as compared with those infected with A/Iran-05 (median difference in evolutionary persistence between serotype A and O lineages of 4.0 years) (fig. 5*a*, supplementary table S3, Supplementary Material online). These findings likely account for the role of large and small ruminant populations as the key host species driving FMDV diversity across the region, and possibly indicative of a relative species-specific variation in transmissibility of FMDV serotypes among host species, albeit space-time heterogeneity in virus sampling from different hosts could have influenced this latter finding.

### Determinants of FMDV Diffusion

A generalized linear model (GLM) parameterized on the discrete phylogeographic inference framework was used to identify likely predictors of FMDV dispersal between countries. Among the 58 predictors included in the analysis (supplementary table S4, Supplementary Material online), 5, 9, and 1 factors yielded statistical support when tested for the A/Iran-05, O/PanAsia-2, and Asia1/Sindh-08 lineages, respectively, with one predictor found to be shared by all lineages (table 2, supplementary fig. S6, Supplementary Material online). As expected, this shared predictor described FMDV diffusion to be strongly correlated between countries that share international borders (shared border: BF > 50), thus identifying geographical contiguity as a significant factor for easing virus movements between countries. Furthermore, these analyses indicated that livestock trade is one of the key drivers for virus dispersal between and within countries of the Western and Southern Asia regions. We found a statistical association between the volume and value of trade in large ruminants and the reconstructed geographic transitions of FMDV lineages, with a stronger supporting evidence reported for A/Iran-05 (head and value of imported large ruminants: BF > 100) in comparison with O/PanAsia-2 (head and value of imported large ruminants: BF = 7.0). Livestock production and population characteristics were also observed to influence the FMDV dispersal from the country of origin, although these indicators affected the dynamics of FMDV lineages differently. The size of the small ruminant population in FMD affected countries was only significantly associated with the rate of regional spread of the O/PanAsia-2 lineage (small ruminant stock: BF = 12.4). In addition, both grassland-based livestock production systems (i.e. agropastoral/pastoral) (LGA: BF > 50) and mixed rainfed systems (i.e. crop-livestock) (MRA: BF = 37.8) of arid and semiarid ecosystems at origin were identified as significant predictors of O/PanAsia-2 virus diffusion. The LGA system is widely distributed across the entire region, whilst MRA is generally present in western Iran and northern Pakistan (Robinson et al. 2011). In the case of A/Iran-05, virus diffusion was inversely associated with the livestock production output at the origin (LPI: BF > 100), likely indicating virus outflow along with livestock movements toward more profitable markets.

**Table 2.** Predictors of FMDV Transmission within the Western and Southern Asia Region Estimated from the Phylogenetic Generalized Linear Model.

FMDV Lineage	Predictor	Coefficient [95% BCI]	Odds Ratio	Inclusion *P*	BF
A/ASIA/Iran-05	shareBorder	3.23 [2.64 to 3.87]	25.28	1	>100
	sample_Or	1.20 [0.76 to 1.72]	3.32	1	>100
	LPI_Or	−1.35 [−2.19 to −0.70]	0.26	0.94	>100
	valImpMat_LR	0.24 [−3.13 to 3.03]	1.27	0.55	>100
	headImpMat_LR	0.19 [−3.38 to 3.56]	1.21	0.41	>100
O/ME-SA/PanAsia-2	sample_Des	0.75 [−1.37 to 1.93]	2.12	0.87	>100
	geoDist	−1.02 [−2.59 to 1.70]	0.36	0.82	>100
	shareBorder	0.93 [−2.96 to 3.62]	2.53	0.46	>50
	LGA_Or	0.38 [−3.38 to 3.49]	1.46	0.40	>50
	MRA_Or	0.27 [−3.66 to 3.58]	1.31	0.24	37.80
	stockSR_Or	0.16 [−3.59 to 3.95]	1.17	0.10	12.41
	headImpMat_LR	0.01 [−3.74 to 3.89]	1.01	0.06	7.31
	valImpMat_LR	0.05 [−3.71 to 3.89]	1.05	0.06	6.97
	sample_Or	0.04 [−3.77 to 3.87]	1.04	0.06	6.92
Asia1/ASIA/Sindh-08	shareBorder	1.51 [−2.34 to 4.12]	4.53	0.66	>50

Note.—Predictors are reported in decreasing order of their estimated Bayes Factor (BF). Coefficients are expressed as log base 10. Details of each of the predictors used in the analysis are described in the supplementary table S4, Supplementary Material online.

The phylogenetic GLM analysis revealed no further statistical evidence for the contribution of any of the other potential covariates tested to the regional dissemination of FMDV, such as economic indicators of countries, agricultural land uses, trade in meat, and price differences in the livestock commodities trade. However, we found the size of the sample collected at origin for A/Iran-05 (BF > 100) and at destination for O/PanAsia-2 (BF > 100) having an impact on the prediction of virus transitions.

## Discussion

Here we provide the first comprehensive retrospective of endemic FMD dynamics in one of the most epidemiologically active regions of the world, which corresponds to the full extent of endemic Pool 3 (Paton et al. 2009). By integrating data from ∼2,500 FMDV sequences coding for the VP1/1D protein with relevant geographic, host, and epidemiological covariates in a phylodynamic framework, we document the evolutionary processes that have driven the emergence, migration, and expansion of FMDV strains circulating within- and between-specific regional ecosystems of Western and Southern Asia.

The reconstructed network of spatial diffusion provides evidence about how emergence and dispersal of FMDV lineages follow a stochastic dynamic of alternating waves of different serotypes, a pattern that has been previously observed in eastern Africa (Casey-Bryars et al. 2018). Analyses of the evolutionary origins of tree-trunk lineages suggest that persistent and extended virus transmission is maintained within the Southern Asia ecosystem that ignites the circulation of viruses more widely across the endemic Pool 3. Specifically, the dynamics of FMDV flow identified Afghanistan, Pakistan, and Iran not only as primary conveyors of infections but also as important sites to generate new evolutionary substrate. Other countries contribute to the diffusion of viruses at the subregional level or act as cul-de-sac systems (such as Turkey), where FMDV lineages may persist and continue to evolve even to sometimes establish new variants, but further migration and spread of these tend to be epidemiologically confined to a local level. The region enclosed within Western and Southern Asia represents a geographical crossroads of livestock movement between Europe, Asia, and Africa. Areas of continuous high-density livestock populations between the Mediterranean Basin and Southern Asia south of the Caspian Sea (termed the “Eurasian ruminant street” [Slingenbergh et al. 2004]) creates an important corridor for the spread of pathogens (Wint 2003), an epidemiological pattern that was also recorded during the rinderpest era (Roeder et al. 2006). In this study, the high degree of spatial diffusivity for FMDV type O, A, and Asia1 lineages in a westerly direction provides further support to the role of host population continuity in ecosystems that sustain epidemiological links across the region. In this context, the Southern Asia ecosystem represents a “mixing vessel” that maintains FMD viruses prior to their movement via livestock trade pathways, primarily westwards to West Eurasia and further into North Africa, but also in a more limited fashion to the North into the Central Asian region.

This study demonstrates that the evolutionary dynamics of FMDVs circulating within endemic Pool 3 are governed by a complex process characterized by competition between geographically coexisting virus strains. Analyses of genetic differentiation of type O and A lineages revealed continuous and parallel evolution, leading to high degree of variability between variants that characterized spatially heterogeneous and temporally varying habitat patches. These results support the hypothesis of a source-sink model of FMDV ecology in which new variants sequentially replace viruses that previously occupied similar ecological niches. The observed periodic establishment of new FMDV sublineages is hypothesized to be influenced by the effect of selection and asymmetric (nonrandom) migration from the source population in Southern Asia, where high infection rates drive the long-term evolution of FMDV. The high transmissibility of FMD coupled with areas where high densities of susceptible host species exist, facilitate the rapid diffusion of these newly emerging virus strains across a wide geographical landscape, and contribute to the mixing of distinct FMDV sublineages.

Pre-existing immunity and virus–host fitness are considered to be important factors that influence the dispersal and persistence of virus populations in source-sink systems, by mediating habitat quality and suitability (Parrish et al. 2008; Rouzine and Rozhnova 2020). In this study, evolutionary estimates of cross-species transmission are indicative of fitness preferential of FMDV serotypes to specific hosts: type O viruses were found to have longer waiting time for virus transfer in small ruminants as opposed to type A lineages, which were shown to have longer duration in cattle. This signal of evolutionary adaptation in host species could support the hypothesis that different serotypes have “permissive evolution” within specific host species that might influence virus diffusion in different ecological systems. It is likely that selection and diversification of FMD viruses driven by preferential transmission in different host populations facilitate viral niche differentiation, thus resulting in an epidemiologically balanced coexistence of multiple lineages evolving from different FMDV serotypes. Therefore, distinct FMDV strains may coexist with their relative frequencies with the potential to invade new geographical areas leading to parallel evolution, and thus creating multiple pseudoindependent subpopulations with distinct spatial structures. In this epidemiological context, every FMDV population undergoes constant evolution, even though the degree of this process might vary from one strain to another. If the epidemiological conditions present in a newly invaded ecosystem are favorable and sufficiently varied, each FMDV population will further expand their own evolutionary path superseding previously circulating strains by source-sink dynamics of virus replacement.

It should be emphasized that most inference-based conclusions drawn here on FMDV ecology depend on complex, multifactorial evolutionary, and population-level processes (such as within-host dynamics and immunological profile of host species, mechanisms of viral fitness, and environmental effects on susceptibility to infection, among others), which are not (and cannot be) fully captured from the analyses undertaken in this study. However, by reconstructing the phylodynamics and phylogeography of three of the most evolutionary diverse and geographically persisting FMDV lineages of endemic Pool 3, we have started to contribute the process of dissecting the full ecological model of FMDV evolution in endemic systems.

Further supported by the results of the phylogenetic GLM analyses, we hypothesize that successful fixation of newly emerging FMDV variants depends not only on their genotypic features but also on the specific ecosystem in which they interact, with virus–host interactions and ecological factors providing relative permissive conditions for virus evolution and further generating competitive interactions among FMDV strains. Despite constant political and cultural upheaval that makes the study region increasingly fragmented, countries of Western and Southern Asia are interconnected on both ecological and epidemiological basis, from livestock farming practices to dynamics of livestock trade (Shimshony and Economides 2006; Di Nardo et al. 2011); providing an underpinning mechanism by which newly emerged FMD viruses can spread via transboundary movements. Our findings suggest that both movements of existing FMDV strains and introduction of new variants across the study region are mediated by host characteristics and their distribution, livestock production systems and trade. Covariates of FMDV transmission associated with agropastoral/pastoral (livestock only) and crop-livestock production systems of arid/semiarid ecosystems were identified as origin effect, in addition to the rate of virus diffusion influenced by the volume and value of trade in cattle and the size of a country’s small ruminant population. With our analyses, we did not account for livestock trade and cross-border movements that occur through informal channels, which represent an important activity in the region (Grace and Little 2020), thus increasingly adding to the risk of FMDV diffusion through livestock movement here identified by only examining official data.

Findings from this study highlight that FMD endemic areas characterized by large populations of small ruminants have a direct impact on the rate of O/PanAsia-2 spatial diffusion, which when linked with the observation of a longer duration of O/PanAsia 2 persistence in these species, provide evidence of how small ruminant populations might play an important role in the epidemiological dynamics of the type O PanAsia-2 FMDV lineage, likely contributing to the endemic maintenance of these viruses. The fact that FMD most often assumes a subclinical condition in sheep and goats (Kitching and Hughes 2002) makes these host species a potential hidden-source of infection for cattle in mixed livestock systems and further increasing the risk of virus dissemination through animal movement and international trade. Seasonal transhumance and nomadic herding of small ruminants are widely practised within the region, despite an increasing transformation into mixed crop-livestock production systems (van de Steeg and Tibbo 2012). In addition, imports of live sheep account for more than 25% of the total imports for the region, with Islamic religious festivals (such as the Eid al-Adha) accounting for the majority of these figures where millions of animals are moved annually (Sherman 2011). Vaccination within the study region is often used as a tool to prevent economic losses resulting from FMD outbreaks rather than as an effective control measure to reduce virus transmission. In countries characterized by endemic circulation, systematic vaccination is only applied to dairy cattle, whilst sheep, and goats are often either only included in emergency vaccination schemes or excluded from vaccination programs for reducing costs, increasingly making them a potential source of infection.

In this study, inferences on FMDV dynamics are based on genetic analyses of sequences encoding for the VP1/1D region, which covers only ∼8% of the FMDV genome and thus potentially reducing the phylogenetic resolution offered by analyzing the whole-genome sequence (WGS). It should be noted that recombination is not an uncommon event for FMDV, and results generated using WGS data should be treated with caution, especially so when studying endemic systems strongly characterized by cocirculation (and likely coinfection) of distinct FMDV serotypes. In fact, the presence of recombination breakpoints within the VP1 coding region has been shown to be negligible, with previous data suggesting that the exchange of genetic materials is instead more frequent in genome regions outside the FMDV capsid, resulting in mosaic genomes (Bachanek-Bankowska et al. 2018; Ferretti et al. 2018; Lasecka-Dykes et al. 2018). Recombination processes decouple the evolutionary history of different viral genes/proteins, which can similarly lead to the decoupling of their inferred spatial histories. In fact, it has been shown that, in the case of recombination, the WGS does not provide a good summary of the geographic histories of each individual genomic region, whilst the FMDV genome coding for the capsid region (comprising of the structural proteins from VP1 to VP4) have highly coupled histories, both phylogenetically and geographically (Bachanek-Bankowska et al. 2018; Singer et al. 2020). These factors provide confidence in the results based on the use of VP1 sequences to reconstruct spatial migration networks and estimate divergence times and evolutionary rates, although the full capsid region would be expected to provide greater resolution. However, sampling biases represent an important caveat in phylogeographic and phylodynamic studies, with nonheterogeneity and nonconstant effort in sampling likely to affect the ancestral reconstruction of geographic transitions (Baele et al. 2018). Although we acknowledge this may be relevant for the FMDV migration network here reconstructed, we made inferences from a significant number of genetic sequences that cover the full evolutionary and geographical space defined by the timeframe of the study and the geographical extension of the study area. We further attempted to mitigate the effect of biased sampling structures by conditionally accommodating for preferential sampling with the inclusion of sampling-related predictors in the phylogenetic GLM and by using sampling-aware methods for reconstructing viral diversity through time (Karcher et al. 2016). In addition, for inferences on sublineages and host traits we used a more robust method based on the structured coalescent, which is expected to be less sensitive to sampling bias (De Maio et al. 2015). Despite the approaches taken to mitigate these problems, the opportunistic nature of the sampling process with its inhomogeneous structure in both space and time present in the data analyzed could have still affected the results of our study.

In conclusion, our study emphasizes the importance of genomic surveillance of FMDV in endemic regions enhanced by the integration of detailed spatial and epidemiological data, with the potential to elucidate evolutionary drivers of FMD transmission and to provide insights into host population and ecological factors underlying FMDV diffusion. Ultimately, these tools can be translated into actionable results, to guide effective intervention and prevention strategies for the progression toward FMD control in line with global efforts.

## Materials and Methods

### Sequence Data

FMDV sequences encoding the VP1/1D genomic region (of 639 nucleotides in length) used for this study represented FMDV lineages circulating in Western and Southern Asia between 2001 and 2018 and belonging to three different FMDV serotypes: O/ME-SA/PanAsia-2 (*n* = 1,321), A/ASIA/Iran-05 (*n* = 843), and Asia1/ASIA/Sindh-08 (n = 331). The final data set of 2,495 VP1 coding sequences with associated metadata was compiled from the FAO/OIE World Reference Laboratory for FMD (WRLFMD) repository held at The Pirbright Institute, United Kingdom (supplementary table S5, Supplementary Material online). The data set also included publicly available sequences previously deposited in GenBank (Benson et al. 2013) (*n* = 535), as of September 2018. By definition, each sequence represented a single FMD virus isolate collected from clinically affected cases during FMD outbreaks recorded in countries of Western, Central, and Southern Asia (definition of geographic regions was following the M49 standard of the United Nations [UN 1999]). The number of isolates sequenced for each FMDV lineage varied between geographical regions and countries (supplementary table S5, Supplementary Material online): the final data set contained 648 VP1/1D sequences from Western Asia (A/Iran-05 = 286, O/PanAsia-2 = 328, Asia1/Sindh-08 = 34) and 1,720 from Southern Asia (A/Iran-05 = 490, O/PanAsia-2 = 934, Asia1/Sindh-08 = 296), in addition to sequences from other bordering regions of Central Asia (14; A/Iran-05 = 4, O/PanAsia-2 = 9, Asia1/Sindh-08 = 1), Southeastern Asia (14; O/PanAsia-2 = 14), Northern Africa (75; A/Iran-05 = 58, O/PanAsia-2 = 17), and Eastern Europe (24; A/Iran-05 = 5, O/PanAsia-2 = 19). Host species were represented in the data set by the following figures: large ruminants (*n* = 1,765), water buffalo (*n* = 305), small ruminants (*n* = 145), pigs (*n* = 1), and wildlife (*n* = 21). VP1 coding sequences were generated using a standardized technical pipeline for RNA extraction, RT-PCR, and sequencing (Knowles et al. 2016). For each lineage, nucleotide sequence alignments were obtained by back translating AA sequences aligned using MAFFT 7.471 (Katoh and Standley 2013).

### Evolutionary Analysis of Spatiotemporal FMDV Dynamics

Prior to molecular clock analysis, the degree of temporal signal present in the sequence data was evaluated using a root-to-tip regression of genetic distances against sampling time (Rambaut et al. 2016) computed from a maximum-likelihood (ML) tree. The ML tree was parameterized in RaxML 8.2.12 (Stamatakis 2014) using 200 bootstrap replicates under a general time-reversible model (Tavaré 1986) with gamma-discretized among-site rate variation (GTR + Γ), which was determined as the best-fitting nucleotide substitution model by ModelTest-NG 0.1.6 (Darriba et al. 2020). Time-scaled phylogenies were then reconstructed in BEAST 1.10.4 (Suchard et al. 2018). The evolution of FMDV was modeled by parameterizing the process of nucleotide substitution using the GTR + Γ_4_ model, by allowing evolutionary rates to vary across branches according to a log-normal distributed relaxed molecular clock (Drummond et al. 2006), and by using the nonparametric Skygrid coalescent demographic model (Gill et al. 2013) as tree topology prior (setting 100 transition-points for population size changes). Reconstruction of the FMDV dispersal history at the regional level was inferred by defining a nonreversible continuous-time Markov chain (CTMC) model of discrete geographic traits (i.e., countries) (Lemey et al. 2009), which account for variabilities in diffusion rates between locations. The Bayesian stochastic search variable selection (BSSVS) procedure was used to computationally infer ancestral migrations efficiently by determining the network of nonzero transition rates from the CTMC asymmetric matrix (Lemey et al. 2009). A stochastic mapping of FMDV lineage movement events (Markov jumps) and waiting time for transitions between countries (Markov rewards) was further generated by analyzing the posterior inference of the complete Markov jump history through time (Minin and Suchard 2008). The prior for the molecular clock rate was defined by a noninformative CTMC conditional reference prior (Ferreira and Suchard 2008), a truncated Poisson prior was set for the number of nonzero rates, with all the remaining priors left at their default values. The joint posterior estimates were obtained running a Markov chain Monte Carlo (MCMC) for 200 million iterations and recovering samples every 20,000 states. Mixing and convergence of the MCMC chain was assessed using Tracer 1.7.1 (Rambaut et al. 2018), ensuring sufficient sampling was achieved (where the Effective Sample Size was at least 200 and not lower than 500 for each of the posterior parameters). The 9,000 posterior trees resulting following removal of the initial 10% of sampled trees as burnin were used for subsequent analyses. Historical viral population dynamics for each FMDV lineage were reconstructed using the Bayesian Nonparametric Population Reconstruction (BNPR) model (Karcher et al. 2016) as implemented in the R package *phylodyn* (Karcher et al. 2017), which explicitly accounts for preferential sampling of sampled sequences.

### Inference of FMDV Lineages Diffusion in Continuous Space

Auxiliary to the results obtained by the discrete phylogeographic inference method, the multivariate diffusion approach implemented in BEAST 1.10.4 (Lemey et al. 2010; Suchard et al. 2018) was used to reconstruct the dispersal of FMDV lineages in a continuous space. Geographic coordinates were extracted from the exact location of the sample where available; otherwise, the centroid of the lowest level of country administrative divisions was used. Sequence alignments for each of the FMDV lineages were analyzed separately by setting a relaxed random walk model that employs a Cauchy distribution to define branch-specific variation in spatial dispersal rates. Substitution, molecular clock, and coalescent models with their prior specifications were set identically to the initial evolutionary analysis, as defined above. The tree topology was kept constant while estimating the new parameters, using the starting tree provided from the initial evolutionary analysis. An MCMC chain of 200 million of iterations was run for each of the FMDV lineages data set, recovering samples every 20,000 states. Inferences were based on the resulting 9,000 trees after discarding the initial 10% of trees as burnin. The R package *seraphim* (Dellicour et al. 2016) was then used to estimate spatial statistics of virus dispersal using a subset of 100 trees uniformly sampled from the posterior set of reconstructed phylogenies. The weighted dispersal velocity $v_{w}$ and diffusion coefficient $D_{w}$, along with the evolution of the spatial wavefront distance and the dispersal velocity through time were estimated as previously defined (Pybus et al. 2012; Trovao et al. 2015). Global livestock population density raster layers for buffalo, cattle, and small ruminants were sourced from the Gridded Livestock of the World (Gilbert et al. 2018) Harvard Dataverse V3 repository and used as a spatial background layer for maps.

### Analysis of Livestock Production Systems, Economic, and Trade Indicators as Covariates of FMDV Transmission

Patterns of spatial diffusion of FMDV lineages were further examined by assessing livestock population and production systems, commodities prices, economic, and trade indicators as predictors of FMDV transmission potential. A GLM formulation of the CTMC diffusion process (Lemey et al. 2014) was used to model viral diffusion rates as a log-linear function of potential covariates of FMDV transmission, resulting in the combination of those variables that were statistically identified as predictors of FMDV spatial spread. Fifty-eight potential predictors of FMDV diffusion were sourced from the FAOSTAT (FAOSTAT Food and Agriculture Database 2021), UNDP (UN 2020), World Bank (World Bank Open Data 2021), and FAO/ILRI (Robinson et al. 2018) databases to include in the analysis. Records available for each year comprised in the time frame of the study (from 2001 to 2018) for each country and between each pair of countries were extracted. The obtained figures were then averaged over the 17-year period and used as predictors. Data were provided either as directionality matrices between pairs of countries or both as origin and destination data for country-specific indicators, with all covariate measures (except for binary predictors) log-transformed and standardized before inclusion in the GLM. Inconsistencies in country reporting import–export trade data present in some databases (such as the FAOSTAT) forced the inclusion of trade predictors as distinct import and export directionality matrices. The type, list, and source of the variables included in the phylogenetic GLM analysis are provided in supplementary table S4, Supplementary Material online. We tested for correlation between all predictors by estimating the Pearson product-moment correlation coefficient ρ before including them in the analysis. The GLM implementation was further controlled for potential unobserved heterogeneity modeling two-way random effects to account for country-specific variabilities in the assessment of geographical transitions between pairs of countries (Dudas et al. 2017). In addition, variation in sampling magnitude among FMDV lineages sampled from each country traits was explicitly controlled by adding these as additional predictors. A 50% prior inclusion probability was specified on all the indicators. Substitution, molecular clock, and coalescent models with their prior specifications were set identically to the initial evolutionary analysis defined above. The tree topology was kept constant while estimating the new parameters, using the starting tree provided from the initial evolutionary analysis. An MCMC chain of 200 million of iterations was run for each of the FMDV lineages data set, recovering samples every 20,000 states. Inferences were based on the resulting 9,000 trees after discarding the initial 10% of trees as burnin.

### Structured Coalescent Analysis of FMDV Sublineages and Host Diffusion

Phylogenetic inference of population structure was performed using the structured coalescent model approximation implemented in the BASTA 3.0.1 (De Maio et al. 2015) package developed for BEAST 2.6.2 (Bouckaert et al. 2019). For the analysis of host species, we investigated virus diffusion between hosts by defining three subpopulations: large ruminants (here defined only by cattle), small ruminants (comprising both sheep and goat), and buffalo. For the analysis of virus sublineages, subpopulations were set using the FMDV nomenclature defined by the WRLFMD and the OIE/FAO FMD Laboratory Network (Knowles and Samuel 2003). The BASTA model was run using the BSSVS procedure with an MCMC chain of 200 million iterations, sampling every 20,000 states. Analyses were performed on the resulting 9,000 trees after discarding 10% of the chain as burnin.

### Analysis of Natural Selection and Genetic Diversity in FMDV Lineages

We investigated episodic positive or diversifying selection on a per-site basis across the FMDV VP1 coding alignment for each of the FMDV lineages in HyPhy 2.5.18 (Kosakovsky Pond et al. 2020) using Single Likelihood Ancestor Counting (SLAC) (Kosakovsky Pond and Frost 2005), Fast Unconstrained Bayesian AppRoximation (FUBAR) (Murrell et al. 2013), and Mixed Effects Model of Evolution (MEME) (Murrell et al. 2012) methods. AA residues identified under positive selection by at least two of the algorithms used were mapped onto a three-dimensional structure of the capsid for FMDV O/BFS/1860/UK/67 (Protein Data Bank No: 1FOD) using PyMOL 2.5.0a0 open-source (Schrodinger 2019). The genetic diversity of FMDV was estimated using the Rao’s quadratic entropy (Rao 1982) and assessed at both within- (*α*-diversity) and between- (*β*-diversity) sublineage levels.

### Statistical Analysis

Postprocessing of generated results, statistical analyses, and data visualization was performed in R 4.0.2 (R Core Team 2020), and using the R packages *ggtree* (Yu et al. 2017), *ggplot2* (Wickham 2016), *gganimate*, and *tweenr*.

## Supplementary Material

Supplementary data are available at *Molecular Biology and Evolution* online. 

## Supplementary Material

msab172_Supplementary_Data
