# Supplementary material for: Evolutionary and Ecological Drivers Shape the Emergence and Extinction of Foot-and-Mouth Disease Virus Lineages
Source: Mol Biol Evol. 2021 Jun 11;38(10):4346–61. doi: 10.1093/molbev/msab172 (PMC8476141; doi:10.1093/molbev/msab172)
Supplement: msab172_Supplementary_Data [file msab172_supplementary_data.zip › FMDV_MiddleEast_SupplementaryMaterial.docx]

**Supplementary Information**

**Evolutionary and Ecological Drivers Shape the Emergence and Extinction of Foot-and-Mouth Disease Virus Lineages**

Antonello Di Nardo

Luca Ferretti

Jemma Wadsworth, Valerie Mioulet

Boris Gelman, Sharon Karniely

Alexey Scherbakov

Ghulam Ziay

Fuat Özyörük

Ünal Parlak^7^, Pelin Tuncer‐Göktuna

Reza Hassanzadeh, Mehdi Khalaj, Seyed Mohsen Dastoor, Darab Abdollahi

Ehtisham-ul-Haq Khan

Muhammad Afzal, Manzoor Hussain

Nick J Knowles, Donald P King

**Supplementary Figures**

**
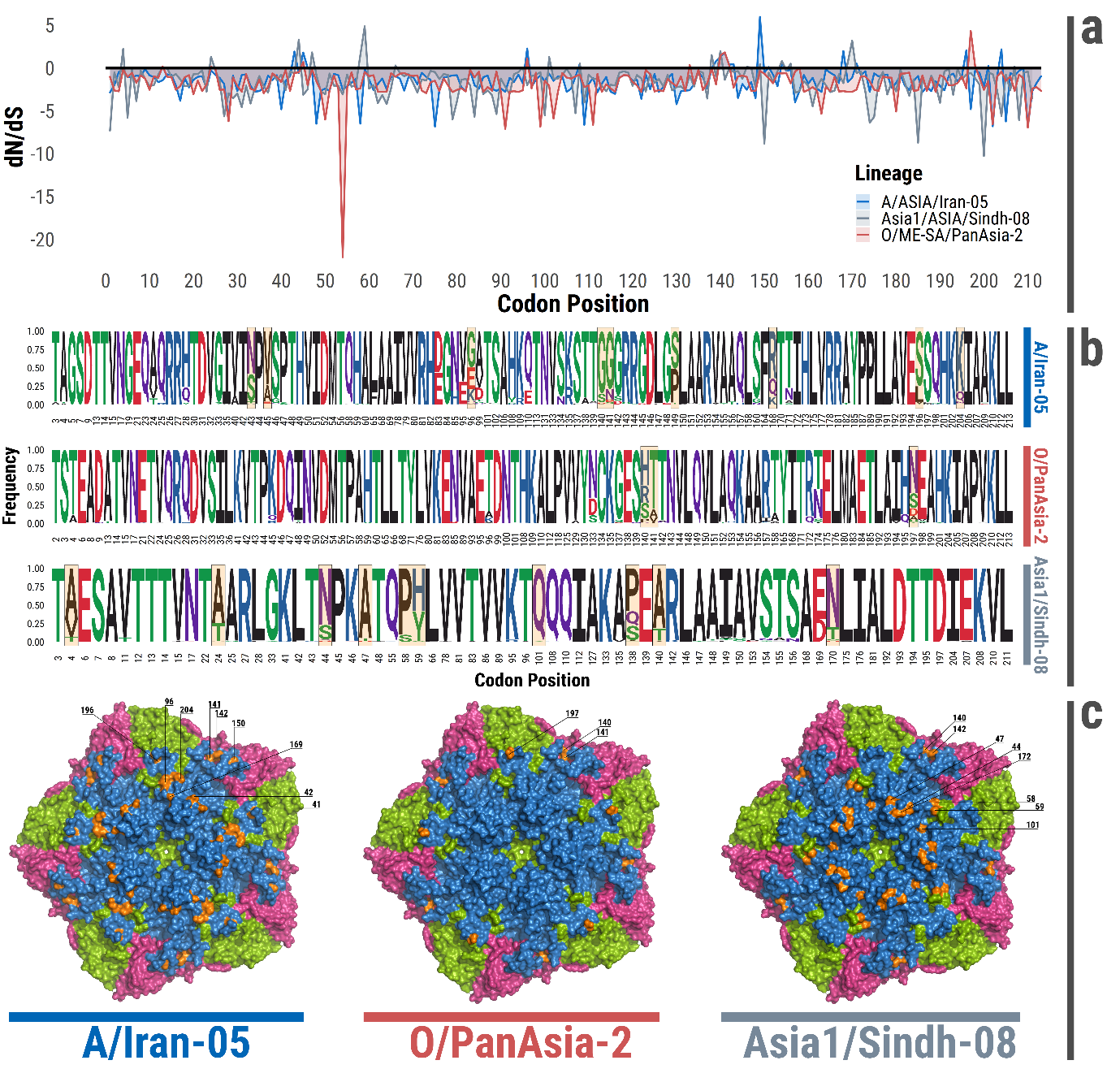
**

**Figure S1 – Selective pressures on the VP1/1D protein in each of the FMDV lineage.** (a) Estimated dn/ds ratios by site in FUBAR (Murrell, et al. 2013). (b) Sequence logo representation of amino acid profile of variable sites of VP1 protein, with orange rectangles identifying residues under positive selection. Amino acids are coloured according to their chemistry. (c) Map of amino acid residues (coloured in orange) identified by analyses in MEME (Murrell, et al. 2012) under positive selection mapped onto the 3D capsid structure of O/BFS 1860/UK/67 FMD virus (Protein Data Bank code: 1FOD). Capsid components of the VP1, VP2 and VP3 are represented in blue, magenta and green, respectively. Codon alignment numbers of positively selected residues are reported and linked with their location in the 3D capsid structure.


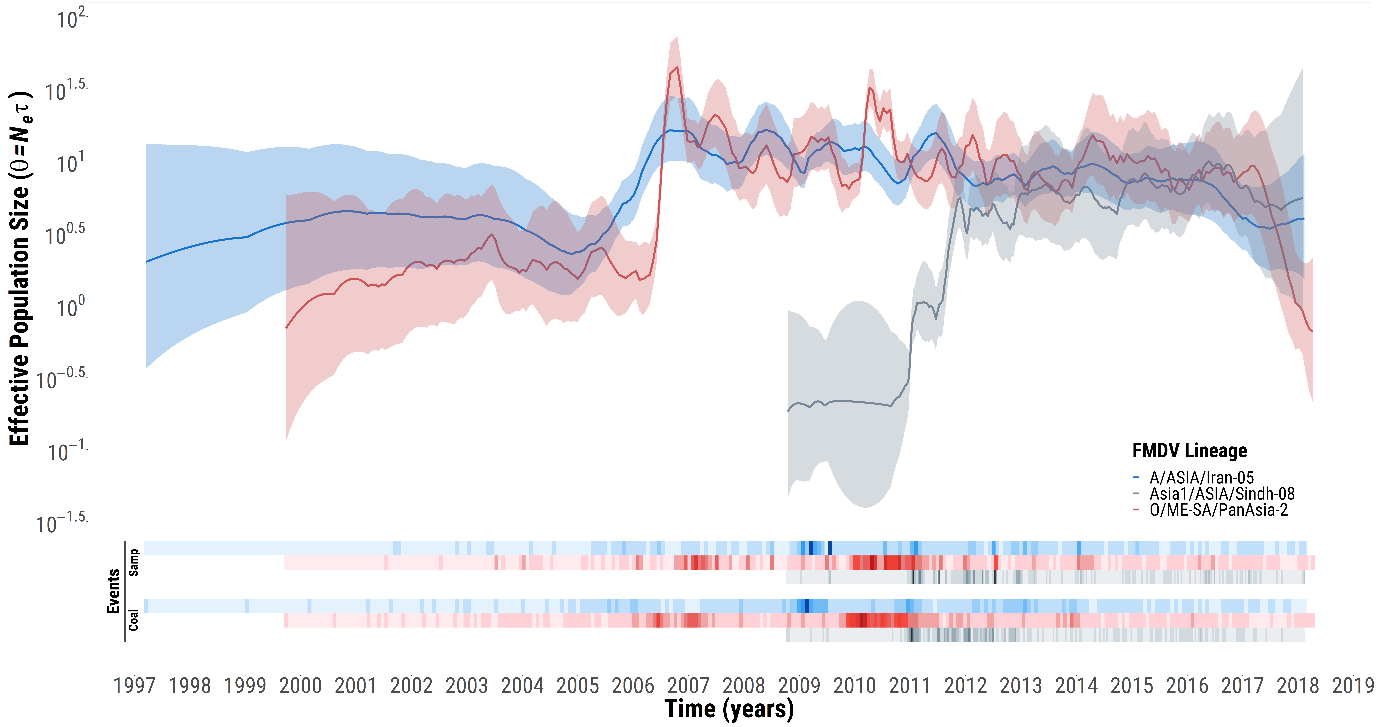


**Figure S2 – Historical trend of viral diversity in FMDV lineages circulating in Western and Southern Asia between 2001 and 2018.** Viral diversity is expressed with the compound estimate $\theta=N_{e}\tau$ of the effective population size as reconstructed using Bayesian nonparametric phylodynamic reconstruction (BNPR) (Karcher et al., 2016). Lines represent median estimates of the effective population size with coloured areas defining the 95% highest posterior density (HPD) region. Upper and lower heatmaps representing, respectively, frequencies of sampling and coalescent events are mapped for each FMDV lineage. The BNPR plots were reconstructed using the data used from this study, and they do not reflect FMDV lineages that were circulating and sampled prior to 2001.


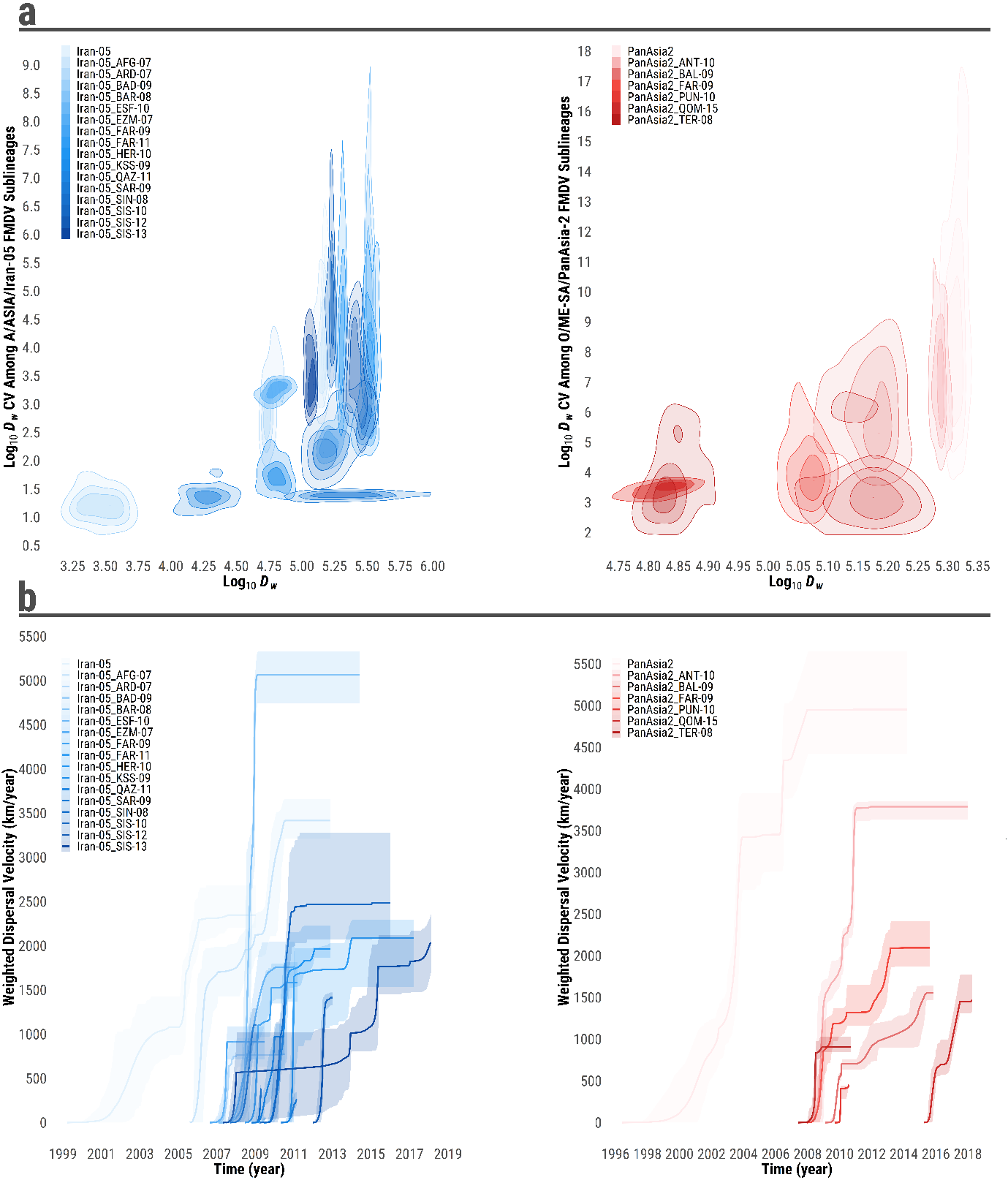


**Figure S3 – Spatial statistics of the dispersal of FMDV sublineages in Western and Southern Asia.** (a) Kernel density estimates of the weighted diffusion coefficient ($D_{w}$) versus the coefficient of variation of $D_{w}$ among branches. The three level contours of decreasing sequential colours identify the 50%, 75% and 95% highest posterior density (HPD) regions. (b) Evolution through time of the FMDV sublineages epidemic wavefront distances from their spatial ancestral origins. The coloured areas represent the 95% credible interval for each of the estimated wavefront spatial location. Median values of all estimated parameters for each FMDV sublineage with corresponding 95% Bayesian credible intervals are reported in Supplementary Table 2.


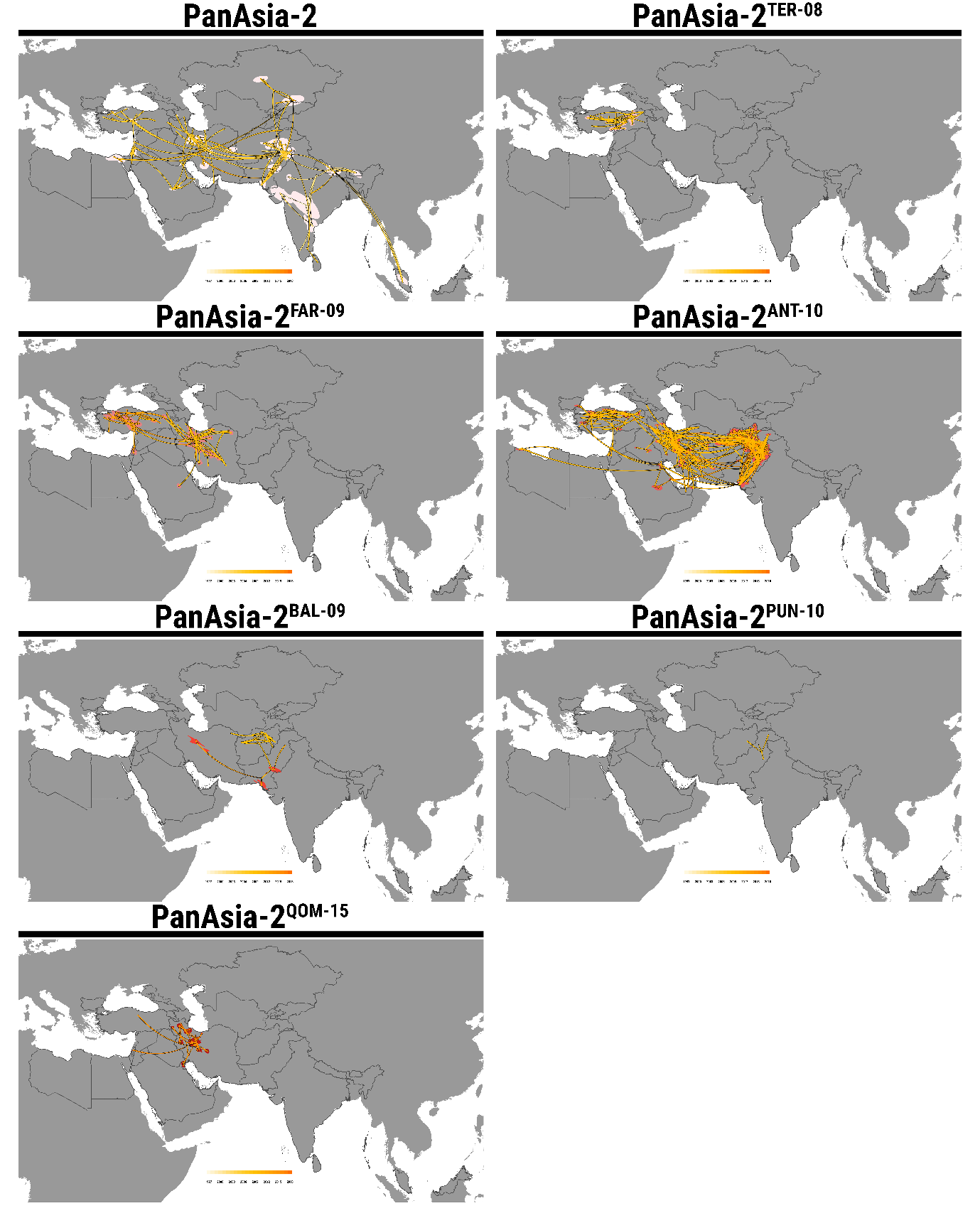


**Figure S4 – Spatiotemporal dispersal of the O/ME-SA/PanAsia-2 FMDV strain within Western and Southern Asia reconstructed for each of its sublineages.** Maximum clade credibility (MCC) trees are mapped along with their 95% highest posterior density (HPD) regions based on 100 trees uniformly sampled from the posterior space of the continuous phylogeographic model results. HPD contours (kernel density estimates) represent statistical uncertainty by time in the estimated locations at the internal nodes.


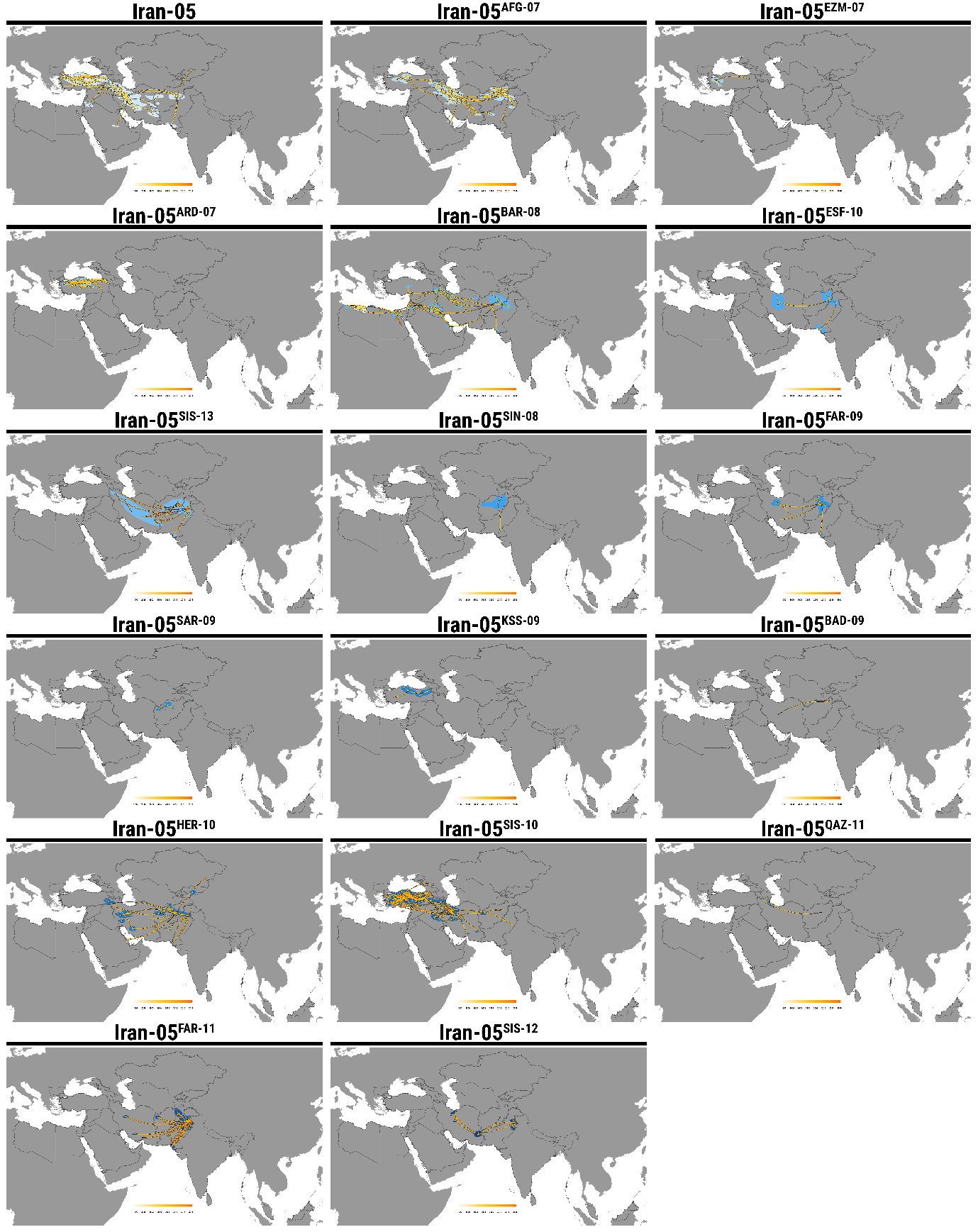


**Figure S5 – Spatiotemporal dispersal of the A/ASIA/Iran-05 FMDV strain within Western and Southern Asia reconstructed for each of its sublineages.** Maximum clade credibility (MCC) trees are mapped along with their 95% highest posterior density (HPD) regions based on 100 trees uniformly sampled from the posterior space of the continuous phylogeographic model results. HPD contours (kernel density estimates) represent statistical uncertainty by time in the estimated locations at the internal nodes.


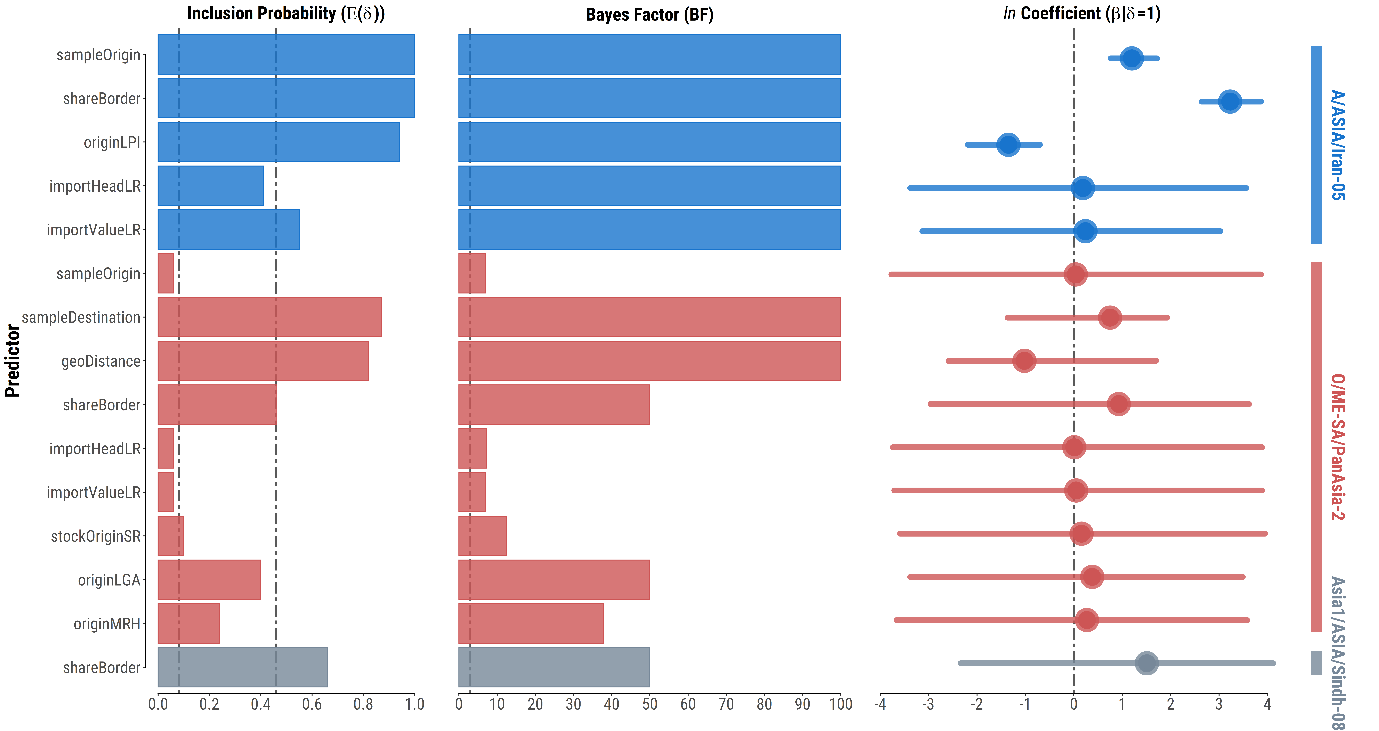


**Figure S6 – Predictors of FMDV spatial diffusion within the Western and Southern Asia region.** Posterior probabilities of predictor inclusion as represented in term of indicator expectation ($E[\delta]$) are provided along with corresponding Bayes factors and estimated coefficients (with associated 95% Bayesian credible intervals). Dash-dot lines report inclusion probabilities at Bayes factors values of 10 and 100. Covariates with an estimated Bayes factor value of ≥3 are reported.

**
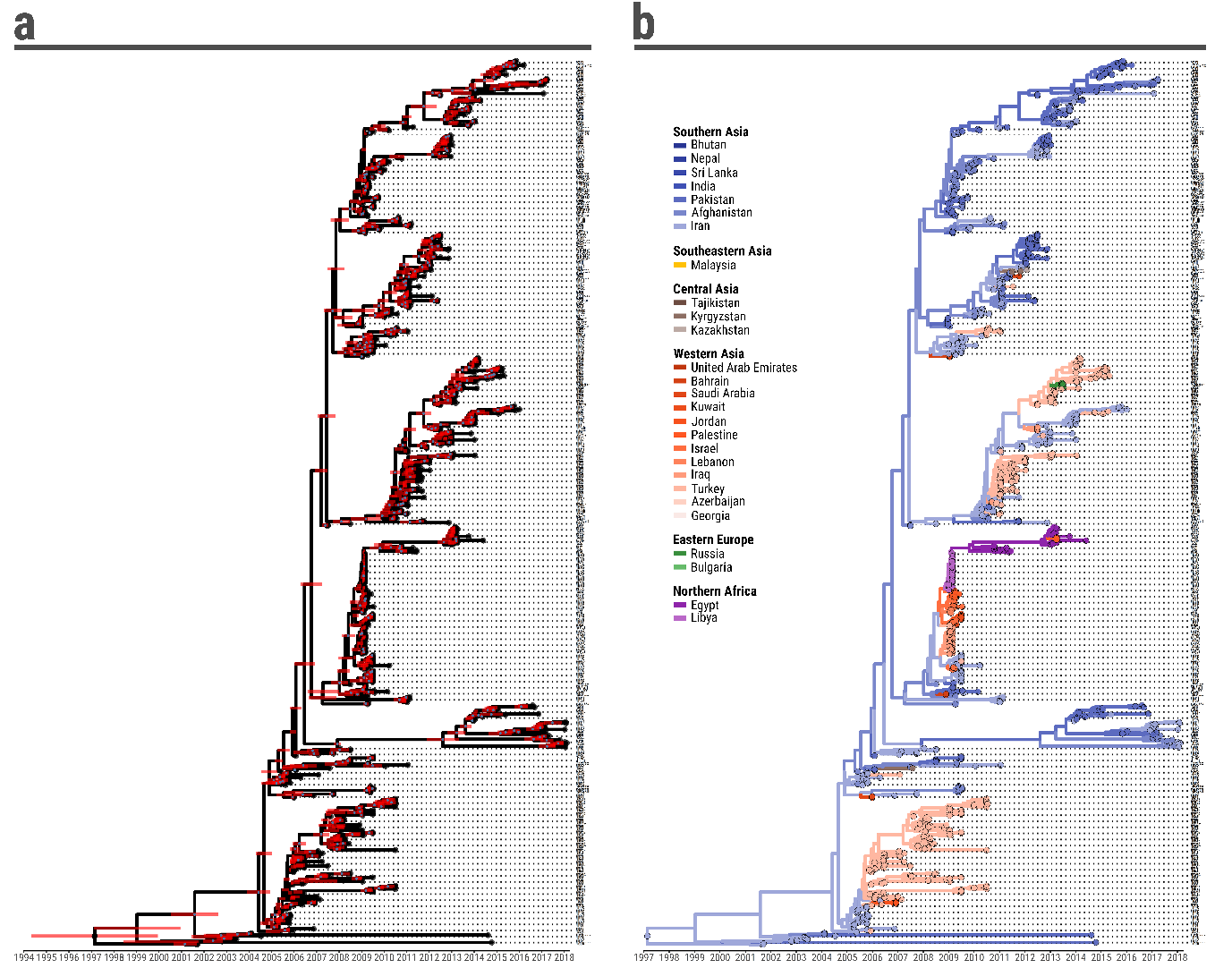
**

**Figure S7 – Molecular clock dating and phylogeography reconstruction of the A/ASIA/Iran-05 FMDV lineage in Western and Southern Asia.** (a) Time-calibrated maximum clade credibility (MCC) tree of n=843 FMDV VP1/1D sequences. Clade support is reported for each internal node associated with a posterior probability of >0.75 (circles coloured in light blue). The 95% Bayesian credible interval reporting uncertainty region in the timing of each ancestral node is represented with red horizontal bars. (b) Spatial-stamped maximum clade credibility (MCC) tree of n=843 FMDV VP1/1D sequences. Branch colours indicate the most probable ancestral location inferred from the discrete state phylogeography analysis.


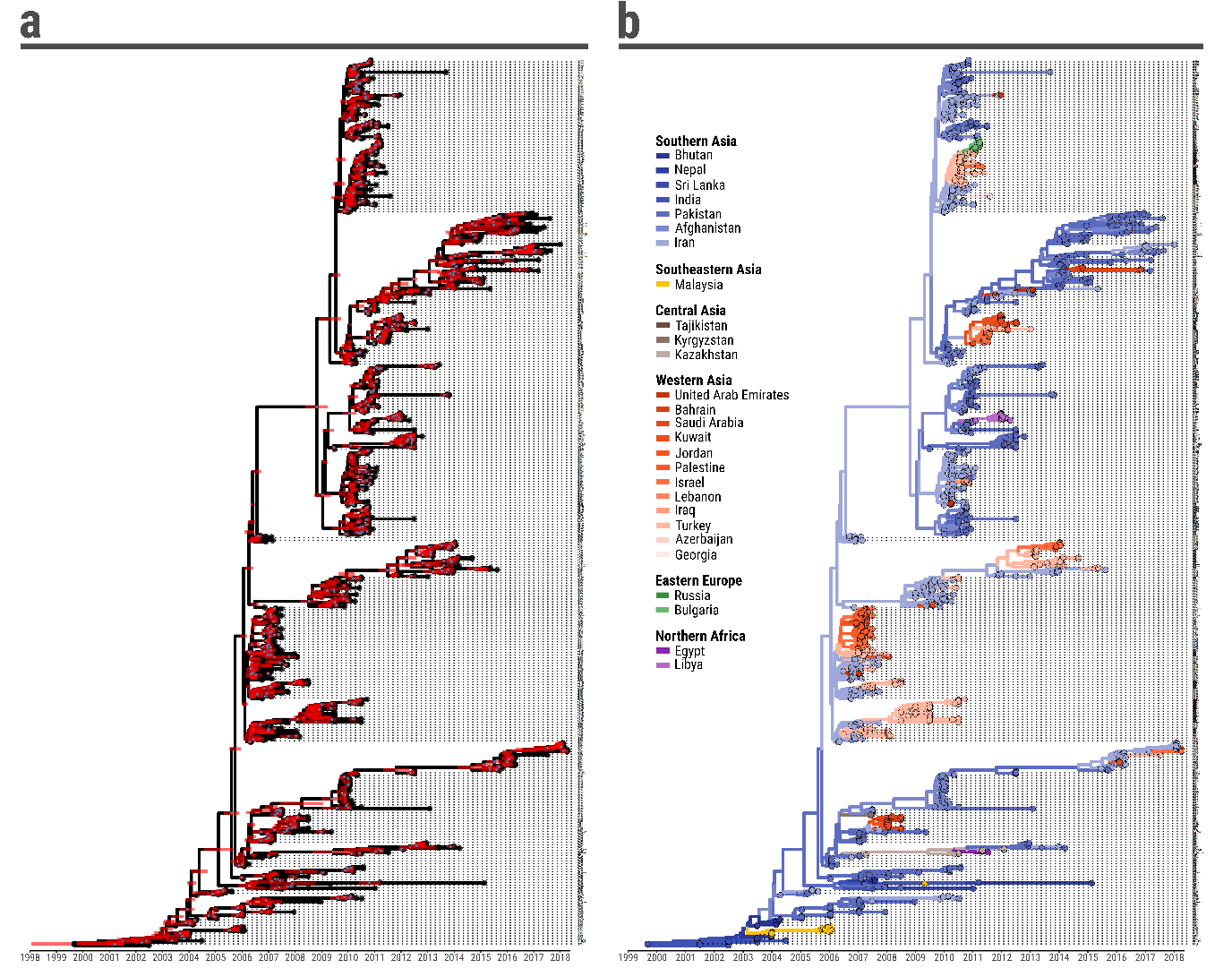


**Figure S8 – Molecular clock dating and phylogeography reconstruction of the O/ME-SA/PanAsia-2 FMDV lineage in Western and Southern Asia.** (a) Time-calibrated maximum clade credibility (MCC) tree of n=1231 FMDV VP1/1D sequences. Clade support is reported for each internal node associated with a posterior probability of >0.75 (circles coloured in light blue). The 95% Bayesian credible interval reporting uncertainty region in the timing of each ancestral node is represented with red horizontal bars. (b) Spatial-stamped maximum clade credibility (MCC) tree of n=1231 FMDV VP1/1D sequences. Branch colours indicate the most probable ancestral location inferred from the discrete state phylogeography analysis.


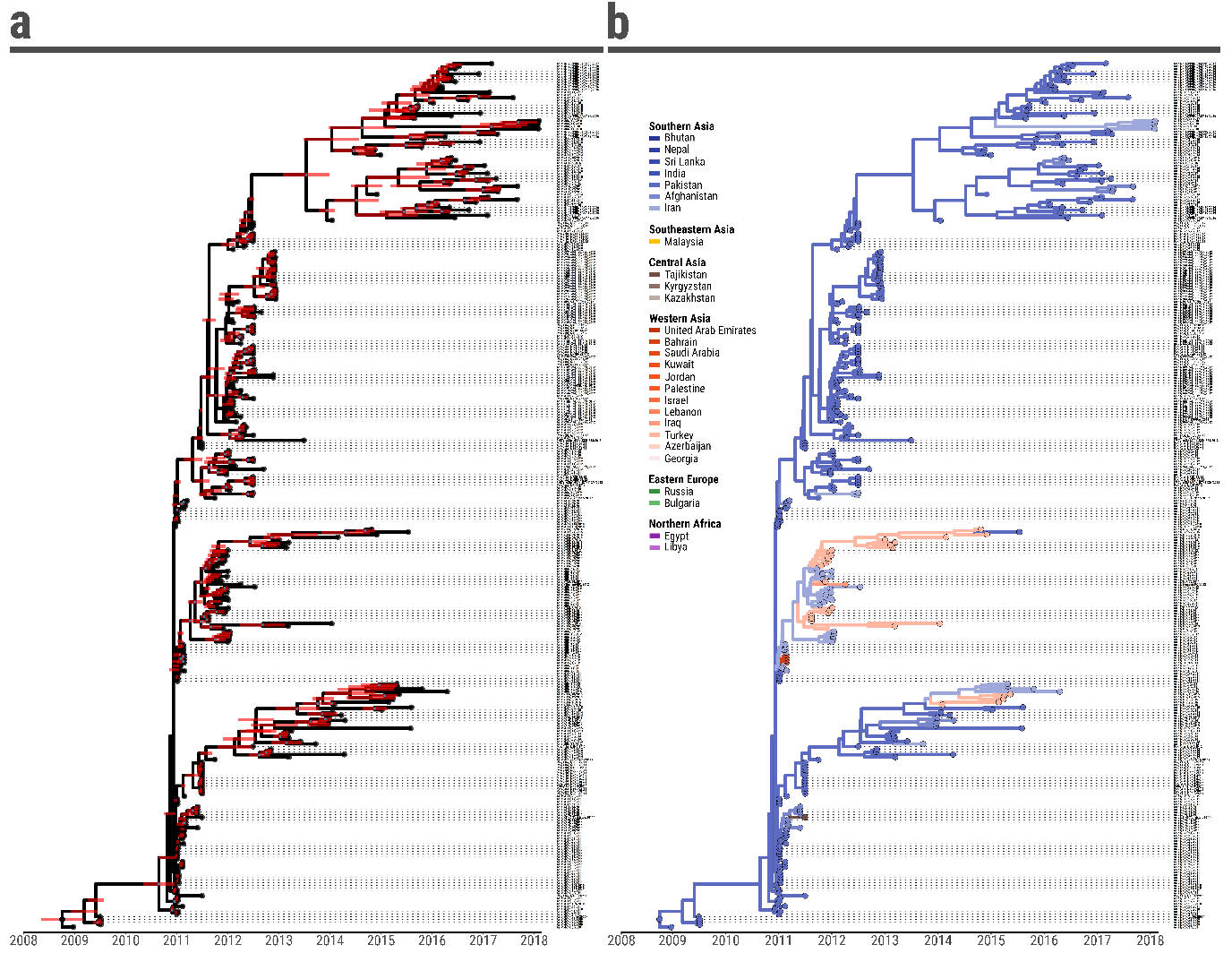


**Figure S9 – Molecular clock dating and phylogeography reconstruction of the Asia1/ASIA/Sindh-08 FMDV lineage in Western and Southern Asia.** (a) Time-calibrated maximum clade credibility (MCC) tree of n=331 FMDV VP1/1D sequences. Clade support is reported for each internal node associated with a posterior probability of >0.75 (circles coloured in light blue). The 95% Bayesian credible interval reporting uncertainty region in the timing of each ancestral node is represented with red horizontal bars. (b) Spatial-stamped maximum clade credibility (MCC) tree of n=331 FMDV VP1/1D sequences. Branch colours indicate the most probable ancestral location inferred from the discrete state phylogeography analysis.

**Supplementary Tables**

**Table S1 – Comparison of selective pressure on the VP1/1D protein estimated at the FMDV lineage level.** Mean posterior values with associated 95% Bayesian credible intervals (BCI) are reported. FMDV sublineages are listed in ascending order by their estimated time of the most recent common ancestor (tMRCA). *Residue sites for Asia1/Sindh-08 and A/Iran05 FMDV lineages have been remapped to the capsid structure of the O/BFS 1860/UK/67 FMD virus (Protein Data Bank No: 1FOD). AA = amino acid.

| **FMDV Lineage** | ***d_N_/d_S_* [95% BCI]** | **No Positive Selected Sites (AA position)** |
| --- | --- | --- |
| A/ASIA/Iran-05 | 0.196 [0.167 – 0.211] | 9 (43, 45, 96, 141, 142, 150, 169, 196, 204)^*^ |
| O/ME-SA/PanAsia-2 | 0.144 [0.127 – 0.158] | 3 (140, 141, 197) |
| Asia1/ASIA/Sindh-08 | 0.183 [0.169 – 0.203] | 10 (4, 24, 44, 47, 58, 59, 101, 140, 142, 172)^*^ |

**Table S2 – Spatial dispersal statistics of FMDV sublineages.** Parameters were estimated from the continuous phylogeographic analyses performed for each of the FMDV lineages, with values reported as posterior median with their associated 95% Bayesian Credible Intervals (BCI). FMDV sublineages are listed in descending order according to their estimated tMRCA (expressed in decimal time unit). tMRCA = time of the most recent common ancestor; tMRS = time of the most recent sample; $v_{w}$ = weighted dispersal velocity (km/year); $D_{w}$ = weighted diffusion coefficient (km^2^/year).

| **FMDV** | | **tMRCA** | **tMRS** | $\boldsymbol{v}_{\boldsymbol{w}}$ **(km/year) [95% BCI]** | $\boldsymbol{D}_{\boldsymbol{w}}$ **(km^2^/year) [95% BCI]** |
| --- | --- | --- | --- | --- | --- |
| Lineage | Sublineage |  |  |  |  |
| Asia1/ASIA/Sindh-08 |  | 2008.757 | 2018.093 | 647.5 [601.3 – 689.1] | 158029.3 [144489.2 – 186222.9] |
|  |  |  |  |  |  |
| A/ASIA/Iran-05 | Iran-05 | 1997.141 | 2009.038 | 780.7 [697.6 – 872.3] | 156010.1 [131794.2 – 181564.5] |
|  | Iran-05^AFG-07^ | 2006.121 | 2012.877 | 799.3 [725.6 – 863.2] | 141515.9 [117776.2 – 165329.7] |
|  | Iran-05^EZM-07^ | 2007.129 | 2009.496 | 383.1 [302.5 – 523.0] | 61995.9 [43423.8 – 93553.0] |
|  | Iran-05^ARD-07^ | 2007.208 | 2010.529 | 549.5 [489.7 – 613.3] | 57289.5 [48396.9 – 78816.0] |
|  | Iran-05^BAR-08^ | 2007.624 | 2014.403 | 1330.5 [1198.9 – 1444.3] | 326125.0 [264876.1 – 387321.6] |
|  | Iran-05^ESF-10^ | 2007.894 | 2011.052 | 625.4 [521.7 – 772.4] | 173300.5 [131260.5 – 242275.8] |
|  | Iran-05^SIS-13^ | 2007.911 | 2018.115 | 456.2 [415.5 – 512.7] | 117939.0 [104528.7 – 159570.0] |
|  | Iran-05^SIN-08^ | 2008.387 | 2009.047 | 1015.4 [596.8 – 1722.5] | 177715.5 [103478.5 – 349016.6] |
|  | Iran-05^FAR-09^ | 2008.896 | 2011.153 | 1198.5 [986.4 – 1450.2] | 316028.6 [231640.7 – 403670.2] |
|  | Iran-05^SAR-09^ | 2009.190 | 2009.301 | 2461.6 [720.6 – 9739.3] | 210388.1 [63356.8 – 970273.6] |
|  | Iran-05^KSS-09^ | 2009.234 | 2010.496 | 695.7 [521.5 – 937.8] | 63980.2 [45104.2 – 116907.3] |
|  | Iran-05^BAD-09^ | 2009.240 | 2009.526 | 392.7 [160.3 – 604.6] | 2827.1 [953.4 – 4876.3] |
|  | Iran-05^HER-10^ | 2009.817 | 2012.888 | 1217.2 [1073.9 – 1356.6] | 337248.1 [288870.8 – 408713.2] |
|  | Iran-05^SIS-10^ | 2009.972 | 2016.014 | 917.3 [858.4 – 988.6] | 170728.1 [149697.4 – 201969.0] |
|  | Iran-05^QAZ-11^ | 2010.697 | 2011.137 | 565.0 [350.8 – 960.4] | 18736.2 [10709.1 – 36997.7] |
|  | Iran-05^FAR-11^ | 2010.859 | 2017.230 | 829.3 [760.9 – 930.6] | 202129.2 [172835.5 – 229566.2] |
|  | Iran-05^SIS-12^ | 2012.201 | 2013.025 | 1404.8 [1114.4 – 1754.4] | 269489.7 [207906.2 – 351966.7] |
|  |  |  |  |  |  |
| O/ME-SA/PanAsia-2 | PanAsia-2 | 1999.669 | 2014.205 | 624.3 [594.5 – 659.4] | 206713.9 [195314.5 – 218649.5] |
|  | PanAsia-2^TER-08^ | 2008.072 | 2009.493 | 535.0 [478.3 – 611.2] | 69168.2 [60296.8 – 82509.0] |
|  | PanAsia-2^FAR-09^ | 2008.486 | 2015.622 | 683.0 [609.9 – 760.9] | 116239.2 [101580.9 – 134852.6] |
|  | PanAsia-2^ANT-10^ | 2008.741 | 2018.005 | 866.7 [833.0 – 902.2] | 194031.0 [185951.3 – 205059.3] |
|  | PanAsia-2^BAL-09^ | 2009.511 | 2012.497 | 803.6 [709.8 – 906.3] | 150080.0 [116694.5 – 181260.4] |
|  | PanAsia-2^PUN-10^ | 2009.909 | 2010.575 | 689.3 [492.8 – 796.5] | 65328.4 [46012.1 – 76744.7] |
|  | PanAsia-2^KAT-15^ | 2015.132 | 2015.132 | - | - |
|  | PanAsia-2^QOM-15^ | 2015.572 | 2018.263 | 840.3 [648.4 – 1015.3] | 145608.0 [100318.8 – 183780.7] |

**Table S3 – Host-specific evolutionary persistence of FMDV lineages.** Evolutionary persistence estimates are expressed as the (median) waiting time in years for a datum FMDV lineage to spread throughout a new host population. The 95% Bayesian credible interval (BCI) for each of the values is reported in square brackets.

|  | **A/ASIA/Iran-05** | **O/ME-SA/PanAsia-2** | **Asia1/ASIA/Sindh-08** |
| --- | --- | --- | --- |
| Buffalo | 6.08 [2.06 – 20.14] | 1.21 [0.91 – 1.69] | 1.35 [0.93 – 2.08] |
| Large Ruminants | 6.99 [6.03 – 7.99] | 2.50 [2.33 – 2.68] | 1.70 [1.45 – 2.01] |
| Small Ruminants | 1.17 [0.57 – 6.76] | 5.19 [2.36 – 23.16] | 0.68 [0.01 – 1.96] |

**Table S4 –** Detailed list of predictors included in the phylogenetic GLM analysis [See enclosed file FMDV_MiddleEast_SupplementaryTable4.xlsx]

**Table S5 –** Detailed list of n=2495 samples included in the study [See enclosed file FMDV_MiddleEast_SupplementaryTable5.xlsx]

**Supplementary Movies**

**Movie S1 – Rendering of the A/ASIA/Iran-05 FMDV lineage phylogeography.** [left panel] Time-calibrated maximum clade credibility (MCC) tree of n=843 FMDV VP1/1D sequences with branches coloured with the most probable ancestral location inferred from the discrete phylogeographic analysis. [top-right panel] Map of the directionality (expressed from the thin to the tick end of Bezier curves) of discrete phylogeographic reconstructed migrations of A/Iran-05 viruses between countries (expressed by their centroids). [middle-right panel] Tree-trunk frequencies of ancestral locations through time of A/Iran-05 viruses as inferred from the discrete phylogeographic analysis. [bottom-right panel] Clade frequencies of ancestral locations through time of A/Iran-05 viruses as inferred from the discrete phylogeographic analysis. Colour scheme for countries is detailed in Fig. 2 and Supplementary Fig. 6. [See enclosed file FMDV_MiddleEast_SupplementaryMovie1.mp4]

**Movie S2 – Rendering of the O/ME-SA/PanAsia-2 FMDV lineage phylogeography.** [left panel] Time-calibrated maximum clade credibility (MCC) tree of n=1231 FMDV VP1/1D sequences with branches coloured with the most probable ancestral location inferred from the discrete phylogeographic analysis. [top-right panel] Map of the directionality (expressed from the thin to the tick end of Bezier curves) of discrete phylogeographic reconstructed migrations of A/Iran-05 viruses between countries (expressed by their centroids). [middle-right panel] Tree-trunk frequencies of ancestral locations through time of O/PanAsia-2 viruses as inferred from the discrete phylogeographic analysis. [bottom-right panel] Clade frequencies of ancestral locations through time of O/PanAsia-2 viruses as inferred from the discrete phylogeographic analysis. Colour scheme for countries is detailed in Fig. 2 and Supplementary Fig. 7. [See enclosed file FMDV_MiddleEast_SupplementaryMovie1.mp4]

**Movie S3 – Rendering of the Asia1/ASIA/Sindh-08 FMDV lineage phylogeography.** [left panel] Time-calibrated maximum clade credibility (MCC) tree of n=331 FMDV VP1/1D sequences with branches coloured with the most probable ancestral location inferred from the discrete phylogeographic analysis. [top-right panel] Map of the directionality (expressed from the thin to the tick end of Bezier curves) of discrete phylogeographic reconstructed migrations of A/Iran-05 viruses between countries (expressed by their centroids). [middle-right panel] Tree-trunk frequencies of ancestral locations through time of Asia1/Sindh-08 viruses as inferred from the discrete phylogeographic analysis. [bottom-right panel] Clade frequencies of ancestral locations through time of Asia1/Sindh-08 viruses as inferred from the discrete phylogeographic analysis. Colour scheme for countries is detailed in Fig. 2 and Supplementary Fig. 8. [See enclosed file FMDV_MiddleEast_SupplementaryMovie1.mp4]

**Supplementary Data**

**Data S1 – Alignment of sequences encoding the VP1/1D region of the FMDV genome for the A/ASIA/Iran-05 lineage (n=843).** [See enclosed file FMDV_MiddleEast_SupplementaryDataS1.fasta]

**Data S2 – Alignment of sequences encoding the VP1/1D region of the FMDV genome for the O/ME-SA/PanAsia-2 lineage (n=1321).** [See enclosed file FMDV_MiddleEast_SupplementaryDataS2.fasta]

**Data S3 – Alignment of sequences encoding the VP1/1D region of the FMDV genome for the Asia1/ASIA/Sinsh-08 lineage (n=331).** [See enclosed file FMDV_MiddleEast_SupplementaryDataS3.fasta]

**Supplementary Results**

**Text S1 – Detailed timeline of spatial diffusion of FMDV lineages evolving and occurring within Western and Southern Asia between 1997 and 2018.**

**A/ASIA/Iran-05 FMDV lineage**

**Ancestral reconstruction and evolution during the initial geographic expansion.** Analysis of the time-stamped tree indicated that the most recent common ancestor (MRCA) of the A/Iran-05 FMDV lineage originated from Iran (PP=0.48) during Mar‑1997 (95% BCI Oct-1993 to Dec-1999), and that viruses derived from this common ancestor started to differentiate into the Iran-05 sublineage from mid-2001 (95% BCI mid-2000 to mid-2002). Continuous evolution that was geographically restricted only to Iran was reconstructed until Apr-2004 (95% BCI Dec-2004 to May-2005), when the first transboundary movement was reported in Afghanistan (95%BCI Jan-2005 to Jun-2005; BF=86.4, PP=0.85). Later in Dec-2005 (95% BCI Nov-2005 to Jan-2006), the A/Iran-05 lineage was found to have also spread from Afghanistan to the neighbouring country of Pakistan (BF>1000, PP=1). Westward movements of viruses from Iran into the Western Asia region were estimated to have occurred in 2005, initially in Turkey during Sep-2005 (95% BCI Jul-2005 to Oct-2005; BF>500, PP=1) and then towards the Persian Gulf in Saudi Arabia during Dec-2005 (95% BCI Nov-2005 to Dec-2005; BF=9.3, PP=0.38). A single limited introduction into Jordan from Turkey (BF=58.2, PP=0.79) was dated Oct-2006 (95% BCI Jul-2006 to Dec-2006). A northerly geographic expansion of A/Iran‑05 was recorded in 2006 when the virus spread to the Central Asia region, with the most likely route of introduction reconstructed from Afghanistan to Kyrgyzstan (BF=30.1, PP=0.66) during Jan-2006 (95% BCI Aug-2005 to Sep-2006).

**Differentiation of new virus sublineages and the Iran-05^AFG-07^ evolution.** With the introduction of the Iran-05 sublineage in previously unaffected areas, especially those of Afghanistan, Pakistan and Turkey, parallel evolution of newly raising FMDV variants were documented with the emergence of the Iran-05^AFG-07^ sublineage, which supplanted the ancestral Iran-05 from the beginning of 2006 (95% BCI late-2005 to mid-2006). Sister clades characterising distinct sublineages evolved within the Turkey phylogeographic clade, the latter originated by a single common virus ancestor introduced from Iran (PP=0.97). Starting from Feb-2007, viruses belonging to three different sublineages were reconstructed as having been co‑circulated in Turkey until mid-2010: the Iran-05^EZM-07^ from Feb-2007 (95% BCI Nov-2006 to May-2007), the Iran-05^ARD-07^ from Mar-2007 (95% BCI Dec-2006 to Jul‑2007), and later the Iran-05^KSS-09^ from Mar-2009 (95% BCI Jan-2009 to Jun-2009). This epidemiological scenario indicates how within-country evolution of FMDV variants in Turkey was the result of significant spatial mixing: Iran‑05^EZM‑07^ was found to diffuse in the Aegean region; Iran-05^ARD-07^ was reconstructed to mainly diffuse within the Marmara, Black Sea and Central Anatolia; Iran-05^KSS-09^ was enclosed within the eastern part of the Black Sea region and Eastern Anatolia region.

Evolution of A/Iran-05 viruses continued within the Southern Asia ecosystem, with virus migration documented within and between Iran, Afghanistan and Pakistan until late 2008, when viruses from Iran (BF>15) were further moved into Israel (Aug-2008, 95% BCI Jun-2008 to Oct-2008), Iraq (Oct-2008, 95% BCI Jul-2008 to Dec-2008) and Bahrain (Oct-2008, 95% BCI Sep-2008 to Nov-2008). Between late 2008 and early 2009, the A/Iran-05 FMDV lineage further expanded its geographic area of activity into the Western Asia countries of Lebanon (Dec-2008, 95% BCI Dec-2008 to Jan-2009) and Palestine (Jan-2009, 95% BCI Dec-2008 to Feb-2008), further in North Africa into Libya (Nov-2008, 95% BCI Oct-2008 to Dec-2008) and later from there into Egypt (BF=229.6, PP=0.94) during Oct-2009 (95% BCI May-2009 to Apr-2010). The FMDV variant (designated as Iran-05^BAR-08^) that characterised the Iran-05 expansion into Western Asia and North Africa was reconstructed to have been evolved from the Iran-05^AFG-07^ sublineage during late-2007 (95% BCI early‑2007 to early-2008) within the Southern Asia epidemiological system (allegedly in Afghanistan, PP=0.77). This sublineage was reported to have been causing infections until May-2014.

From the end of 2009, viruses of the A/Iran-05 FMDV lineage were observed to move within and between Pakistan, Afghanistan, Iran, and Turkey, with only a single introduction from Iran into Iraq (BF>500, PP=1) reported in Sep-2010. During this period the Iran-05^AFG-07^ sublineage evolved and characterised spatially structured circulation of viruses grouping into distinct sister clades, some of which had a very restricted and short evolutionary history (such as the FMDV sublineages designated as Iran‑05^BAD‑09^, Iran‑05^FAR‑09^, Iran-05^ESF-10^, Iran-05^QAZ-11^, and Iran-05^SIS-12^). Starting from Jan-2010 (95% BCI Oct-2009 to Mar-2010), the newly emerging Iran-05^SIS-10^ sublineage was reconstructed to have been originating in Iran (PP=0.80) and spreading into Turkey on at least five occasions, two of which that caused extensive circulation of viruses across the entire country [the first during Aug-2010 (95% BCI Jun-2010 to Oct-2010) and the second in Mar-2012 (95% BCI Dec-2011 to May-2012)]. A single introduction of this sublineage into the North Caucasian and Southern Federal districts of Russia was estimated during Mar-2013 (95% BCI Jan-2013 to May-2015), allegedly by viruses moved from Turkey (BF>500, PP=0.97). No further cases of Iran-05^SIS-10^ infections were reported from the last one detected during Jan-2016 in the East Azerbaijan province of Iran. Differently, the contemporary Iran‑05^HER-10^ sublineage was evolving and circulating between Afghanistan, Pakistan and Iran, with the origin of its ancestor dated back to Oct-2009 (95% BCI Jan-2010 to Aug-2009) and geographically associated with Afghanistan (PP=0.99). Iran‑05^HER-10^ was found to diffuse from Afghanistan to Central Asia into Kyrgyzstan during May-2011 (95% BCI Mar-2011 to Jul-2011) and from the latter into Kazakhstan later in Feb-2012 (BF>500, PP=0.98). Viruses grouping within the Iran‑05^HER-10^ clade were only detected until Nov-2012 when the last cases were described in Pakistan.

**Currently circulating sublineages, the Iran-05^FAR-11^ and Iran‑05^SIS-13^ variants.** The latest reports of A/Iran-05 related viruses circulating within the study region are of two distinct FMDV variants, which group in extant clades directly evolved from the Iran‑05^AFG-07^ sublineage, i.e. the Iran-05^FAR-11^ and Iran‑05^SIS-13^ designated sublineages. FMDV viruses belonging to the Iran-05^FAR-11^ clade originated during late Dec-2010 (95% BCI Oct-2010 to Jan-2011) in Pakistan (PP=0.89) and were found to have been actively spreading within the Punjab and Sindh provinces of Pakistan, in Herat and the north-eastern provinces of Afghanistan, with only two introductions reported in south-east and central Iran during 2014 and 2017. The last virus activity of the Iran-05^FAR-11^ sublineage was detected in Mar-2017 within the Panjshir province of Afghanistan. The more recent Iran-05^SIS-13^ sublineage emerged from Pakistan (PP=0.72) during Aug-2012 (95% BCI Nov-2011 to Apr-2013) and, until now, it has been reconstructed to have been moving across large areas of Pakistan, Afghanistan and Iran, with the latest reports of virus circulation dated back in Feb-2019 from the Sindh province of Pakistan. [supplementary fig. S7a and b, supplementary movie S1].

**O/ME-SA/PanAsia-2 FMDV lineage**

**Evolutionary ancestry and geographical origin.** FMD viruses that were found to evolve and group later into the O/PanAsia-2 FMDV lineage started to emerge in India (PP=0.99) during Feb-1999 (95% BCI Nov-1997 to Feb-2002). This ancestral FMDV strain was estimated to have been circulating within the Indian sub-continent and South-eastern Asia [chronologically, in Nepal from Oct-2002 (95% BCI Apr-2002 to Mar-2003), in Bhutan from Apr-2003 (95% BCI Jan-2003 to Jul-2003) and in Malaysia from Aug-2003 (95% BCI May-2003 to Nov-2003)] until Dec-2003 (95% BCI Aug-2003 to Mar-2004) when the virus was reconstructed to have moved for the first time in an westerly direction into Afghanistan, allegedly via Bhutan (BF=30.6, PP=0.58). Viruses within this lineage were found to have diffused between Afghanistan and Pakistan during May-2004 (95% BCI Jan-2004 to Sep-2004; BF>500, PP=1), and from there into Iran in Jan-2005 (95% BCI Oct-2004 to Apr-2005; BF>500, PP=1). Starting from 2005, there was an increasing incidence of O/PanAsia-2 cases and continuous evolution of viruses directly descending from its MRCA were seen in previously unaffected regions of the Southern Asia countries. This epidemiological scenario led to the differentiation of the PanAsia-2 sublineage, with the MRCA of its clade dated back to Feb-2005 (95% BCI Jul-2004 to Aug-2005).

Extensive transboundary movements of this sublineage to the West, North and South of the Southern Asia region were reported between 2006 and 2007. At least four independent introductions into Turkey of viruses circulating in Iran (BF>500, PP=1) were reconstructed, with the first dated as May-2006 (95% BCI Apr-2006 to Jun-2006). By viruses allegedly moving from Turkey (BF=120.1, PP=0.84), the PanAsia-2 sublineage was estimated of having been introduced into Israel during May-2006 (95% BCI Apr-2006 to Jun-2006), from where it further spread to the Western Asia region into Jordan during Aug-2006 (95% BCI May-2006 to Nov-2006; BF=25.8, PP=0.54) and later once more from Israel (BF=103.8, PP=0.82) into Palestine by Oct-2006 (95% BCI Aug-2006 to Dec-2006).

Southward migrations of PanAsia-2 viruses to countries bordering the Persian Gulf basin was reconstructed to have occurred through three different routes: the first chronologically by movement of viruses from Iran (BF>500, PP=1) into the United Arab Emirates (UAE) (Dec-2006, 95%BCI Nov-2006 to Jan-2007); the second from viruses circulating in Jordan (BF=41.2, PP=0.65) to Saudi Arabia (Mar-2007, 95% BCI Jan-2007 to Apr-2007); the last route characterised by viruses moving again from Iran (BF>500, PP=1) into Kuwait (Jul-2007, 95% BCI Apr-2007 to Sep-2007), from there to Bahrain (Sep=2007, 95% BCI Jun-2007 to Nov-2007; BF=280.9, PP=0.93) and finally again into Saudi Arabia (Dec-2007, 95% BCI Sep-2007 to Feb-2008; BF=463.7, PP=0.95).

During 2007, cases of FMD due to PanAsia-2 were reported in Central Asia, with the phylogeographic analysis supporting two independent introductions from Southern Asia: in Feb-2007 (95% BCI Dec-2006 to Apr-2007) into Kazakhstan from viruses originated in Pakistan (BF=15.3, PP=0.41), and in Apr-2007 (95% BCI Jan-2007 to Jun-2007) into Kyrgyzstan via Afghanistan (BF=4.2, PP=0.16). A further expansion of the PanAsia-2 sublineage geographical extent to the North of the Western Asia region was estimated during Jun-2007 (95% BCI Apr-2007 to Jul-2007) and caused by contemporary viruses circulating in Iran (BF=45.3, PP=0.67) moving to Azerbaijan.

**Virus differentiation and emergence of spatially distinct sublineages.** New FMDV variants characterising sister clades directly evolving from the PanAsia-2 sublineage were independently emerging during 2008 in different areas of the Southern Asia region. After continuous introduction of viruses from Iran (PP=1), the PanAsia-2^TER-08^ differentiated and occurred solely in Turkey from Feb-2008 (95% BCI Oct-2007 to May-2008) until Sep-2010 when the last case was reported in the Elazig province: its geographic extent was found to be limited to the Eastern, South-eastern and Central Anatolia regions. Similarly, ancestral viruses of a divergent FMDV sublineage (designed as PanAsia-2^FAR-09^) were found to be circulating initially within the central-western provinces of Iran (PP=0.99) during Jul-2008 (95% BCI Mar-2008 to Nov-2008), expanding into the northern and southern Iranian provinces and from there this new sublineage moved to Saudi Arabia in Jul-2009 (95% BCI Jun-2009 to Aug-2009). Diffusion of the PanAsia-2^FAR-09^ sublineage to the West into Turkey was reconstructed in at least four occasions, with the initial introduction dated Mar-2010 (95% BCI Dec-2009 to May-2010). During Jan-2013 (95% BCI Aug-2012 to Jun-2013) PanAsia-2^FAR-09^ infections were estimated to have been further affecting Israel and being caused by viruses that were closely related with those circulating in Turkey (PP=0.96). Palestine also reported cases later in Dec-2013 that were linked with the outbreak in Israel (PP=0.81). The last case of FMDV host infected by the PanAsia-2^FAR-09^ was reported from the Tehran province of Iran during Aug-2015.

Reports of a new sublineage (designated as PanAsia-2^PUN-10^) originated from viruses circulating in Pakistan (PP=0.99) during Mar-2008 (95% BCI Aug-2007 to Oct-2008) were started to be documented in early-2009. However, infections caused by viruses within this sublineage were restricted only to the Punjab province of Pakistan where outbreaks lasted until Aug-2010, with a single case reported from the Kabul province of Afghanistan during Jan-2010.

**The wide geographic expansion of the PanAsia-2^ANT-10^ variant.** Starting from the end of 2008, viruses directly descending from the PanAsia-2 sublineage and circulating in central Iran (PP=0.62) during Oct-2008 (95% BCI May-2008 to Mar-2009) were evolved into a new FMDV variant, that was later designated as the PanAsia-2^ANT-10^ sublineage. This sublineage has been estimated as the most active of the O/PanAsia-2 derived strains, and further characterised by the largest geographic extension of reported cases. However, spatially mixing subpopulations of viruses evolving within the PanAsia‑2^ANT‑10^ clade were also described to migrate through diverse routes across the study area. The initial expansion of the PanAsia^ANT-10^ sublineage out of Iran was reconstructed starting from Feb‑2009 (95% BCI Dec-2008 to Mar-2009) with at least five distinct introductions into Afghanistan occurring during 2009. These viruses causing infections in Afghanistan were found to have been mainly circulating within the North/Northeast of the country and across the Herat province in the West, and being regularly moved to the East into Pakistan starting from Jun-2009 (95% BCI May-2009 to Jul-2009). Most of PanAsia^ANT-10^ movements out of Pakistan were reconstructed to the West into the Western Asia region and Persian gulf: in at last five occasions geographic transitions were recorded from Pakistan to Iran starting from Sep-2010 (95% BCI Jul-2010 to Dec-2010); twice into Bahrain, initially during 2011 and then in Jul-2014 (95% BCI Mar-2014 to Sep-2014; BF=561.1, PP=0.96), where viruses moved further into Saudi Arabia during Jul-2016 (95% BCI Feb-2016 to Oct-2016; BF=463.7, PP=0.95). Cases of PanAsia^ANT-10^ detected in Tripolitania, Libya, at the end of 2010 were further linked with viruses circulating in Pakistan and reconstructed to have been introduced on Nov-2011 (95% BCI Oct-2010 to Dec-2010; BF=8.1, PP=0.27), with the last report of PanAsia^ANT-10^ in Libya dated at Apr-2012.

Further expanding to the West and South of the Southern Asia region, PanAsia^ANT-10^ viruses were found to move from Iran into different countries of the Western Asia region, to the Persian Gulf, including a single introduction to the North in Georgia during Aug-2011 (BF=7.8, PP=0.26). Chronologically, cases of PanAsia^ANT-10^ infections were recorded within the Persian Gulf region starting from Jul-2009 (95% BCI Jun-2009 to Aug-2009), when viruses from Iran (PP=1) were firstly introduced into Saudi Arabia. Later during Feb-2010 (95% BCI Dec-2009 to Mar-2010) and further in Dec-2011 and Jan-2013 (95% BCI Nov-2011 to Jan-2013), viruses closely related to Iranian isolates collected from the Yazd and Fars provinces were also detected in UAE. Similarly to the spread that occurred during 2007, virus migration along the route from Iran into Kuwait was again reconstructed during Dec-2010 (95% BCI Sep-2010 to Jan-2011), and from there subsequently into Israel (Feb-2011, 95% BCI Dec-2010 to Mar-2011), Bahrain (Feb-2012, 95% BCI Jan-2012 to Mar-2012) and Saudi Arabia (May-2012, 95% BCI Mar-2012 to Jul-2012).

The initial circulation of PanAsia^ANT-10^ in Turkey was reconstructed as early as Feb-2010 (95% BCI Jan-2010 to Mar-2010), with an estimate of at least five distinct introductions of viruses of Iranian origin that expanded into several regions of Turkey, including the Eastern Anatolia, Marmara, Mediterranean, Black Sea, and Aegean. The last movement from Iran was dated Jan-2015 (95% BCI Oct-2014 to Mar-2015). Multiple geographic transitions of viruses out of Turkey were reconstructed to have been linked with distinct FMDV subpopulations evolved within the country: viruses reported from the Marmara region in Feb-2010 and originated from Iran were estimated to have been moved into Europe in Bulgaria during Nov-2011 (95% BCI Sep-2010 to Dec-2010), and related viruses were further found to move into Israel during Feb-2011 (95% BCI Dec-2010 to Mar-2011); different PanAsia^ANT-10^ viruses introduced from Iran in early-2012 and circulated in Turkey until 2014 (PP=0.96), where also reported causing infections in Israel from Jan-2013 (95% BCI Aug-2012 to Jun-2013) and from there moved into Palestine during late-2013.

The current active subclade of the PanAsia^ANT-10^ sublineage comprises viruses circulating within the Southern Asia region and evolved from a single common ancestor originated in Pakistan (PP=0.89) during May-2010 (95% BCI Feb-2010 to Aug-2010).

**PanAsia-2^QOM-15^ evolution and recent virus activity.** Directly evolving from PanAsia-2 ancestors circulating in Afghanistan (PP=0.95), a new O/PanAsia-2 variant later designed as the PanAsia-2^BAL-09^ sublineage emerged from May-2009 (95% BCI Nov-2008 to Sep-2009) in the Balkh and Samangan provinces. This sublineage was only detected in Afghanistan, with only a single introduction reported from the Razavi Khorasan province of Iran in Feb-2010. Viruses grouping into the PanAsia-2^BAL-09^ clade were reconstructed to have subsequently moved from Afghanistan (PP=0.99) to Pakistan, evolving within its Punjab province (PP=0.85) between late 2011 and 2012, and transitioning from there to the Sindh province where a new daughter FMDV variant (designated as PanAsia-2^QOM-15^) started to differentiate during Sep-2014 (95% BCI Feb-2014 to Feb-2015). The PanAsia-2^QOM-15^ sublineage was later introduced in Iran during Feb-2015 (95% BCI Oct-2014 to May-2015), from where subsequently moved to Kuwait (Dec-2015, 95% BCI Nov-2015 to Jan-2016; PP=1), Turkey (Jul-2017; PP=0.99) and Israel (Feb-2018, 95% BCI Dec-2017 to Apr-2018; PP=1).

The PanAsia-2^ANT-10^ and PanAsia-2^QOM-15^ are the epidemiologically active variants of the O/PanAsia‑2 lineage currently causing infections across the study region, with the latest reports of cases from Iran and Israel dated Jan-2018 and May-2019, respectively. [supplementary fig. S8a and b, supplementary movie S2].

**Asia1/ASIA/Sindh-08 FMDV lineage**

**Emergence of a new Asia1 lineage.** Viruses ancestral to the Asia1/Sindh-08 FMDV lineage were reconstructed to have evolved and circulated in Pakistan (PP=0.99) starting from Sep-2008 (95% BCI Mar-2008 to Dec-2008). Cases of both water buffalo and cattle infected by Sindh-08 strains were initially reported only from the Sindh province until Dec-2010 when the virus was recorded to have been moved to the North of the country into the Punjab province. Transboundary migrations of this FMDV lineage from Pakistan (BF>500, PP=1) into the West of the Southern Asia region were inferred by the phylogeographic analysis starting from the beginning of 2011: the first chronologically in Iran during Jan-2011 (95% BCI Nov-2010 to Jan-2011), and at least in two occasions in Afghanistan during late Jan-2011 (95% BCI Dec-2010 to Feb-2011). During the same period, the Asia1/Sindh-08 lineage was further found to have been expanding its geographic area of activity outside of the Southern Asia region, with transitions reconstructed to the South by viruses migrating from Iran into Bahrain in Feb-2011 (95% BCI Jan-2011 to Feb-2011; BF=84.1, PP=0.94), and further recording at least three introductions to the West in Turkey with the initial estimated around Jul-2011 (95% BCI May-2011 to Jul-2011; BF>500, PP=0.99) in the Eastern Anatolia region. A single movement to the North was also reported in Jul‑2011, when the virus was found to have been introduced in Tajikistan from Afghanistan (BF=10.5, PP=0.66). During Apr-2012 infections attributed to the Asia1/Sindh-08 and caused by viruses originated from Iran (BF=25.0, PP=0.82) were further reported from Iraq. Following this increased geographic expansion out of Southern Asia, between 2012 and 2014 viruses belonging to the Asia1/Sindh-08 FMDV lineage were limited in their circulation within Southern Asia, with multiple geographic transitions reconstructed between Pakistan and Iran, and between Pakistan and Afghanistan. New introductions into the Eastern Anatolia region of Turkey were detected during Jan-2014 (95% BCI Dec-2013 to Feb-2014) and Oct-2014 (95% BCI Mar-2014 to Mar-2015): these viruses were once again linked to ancestors previously circulating in Iran.

**Recent reports of Asia1/Sindh-08 and current geographic area of virus circulation.** From 2104 onwards viruses circulating within the study areas were found to group in a single subclade of which common ancestor evolved and originated from the Punjab region in Pakistan during Jan-2012 (95% BCI Nov-2011 to Feb-201) and until 2016 were only detected in Pakistan. Reports of cases due to this strain were reconstructed in Afghanistan starting from Mar-2016 (95% BCI Dec-2015 to May-2016) and in Iran from Feb-2017 (95% BCI Jul-2016 to Sep-2017).

The latest cases of Asia1/Sindh-08 FMDV virus infections that have been recently reported were identified between January and April 2019 in both water buffalo and cattle species from several regions of Pakistan. [supplementary fig. S9a and b, supplementary movie S3]
